# Rational Selection of Cyanines to Generate Conjugate Acid and Free Radicals for Photopolymerization upon Exposure at 860 nm

**DOI:** 10.1002/anie.202108713

**Published:** 2021-10-19

**Authors:** Qunying Wang, Sergey Popov, Alfred Feilen, Veronika Strehmel, Bernd Strehmel

**Affiliations:** ^1^ Department of Chemistry and Institute for Coatings and Surface Chemistry Niederrhein University of Applied Sciences Adlerstrasse 1 47798 Krefeld Germany; ^2^ Spectrum Info Ltd. Murmanskaya 5 02094 Kyiv Ukraine; ^3^ Easytech GmbH Pascalstrasse 6 52076 Aachen Germany

**Keywords:** cyanine, fluorescence, molecular engineering, near-infrared, photopolymerization

## Abstract

Different cyanines absorbing in the NIR between 750 and 930 nm were applied to study the efficiency of both radical and cationic polymerization in combination with diaryliodonium salt. Variation of the connecting methine chain and structure of the terminal indolium moiety provided a deeper insight in the structure of the cyanine NIR‐sensitizer and the efficiency to generate initiating radicals and conjugate acid. Photophysical studies were pursued by fluorescence spectroscopy providing a deeper understanding regarding the lifetime of the excited state and contribution of nonradiative deactivation resulting in generation of additional heat in the polymerization process. Furthermore, electrochemical experiments demonstrated connection to oxidation and reduction capability as influenced by the structural pattern of the sensitizer. LC–MS measurements provided a deeper pattern about the photoproducts formed. A nonamethine‐based cyanine showed the best performance regarding bleaching in combination with an iodonium salt at 860 nm.

## Graphical abstract

Substitution of cyanines with different terminal indolium groups and distinct polymethine chain directs the excited state either to deactivation by internal conversion (IC) or photochemistry resulting in the generation of initiating radicals for free‐radical polymerization and conjugate acid for cationic polymerization and self‐polyaddition of vinyl ether **M1**.

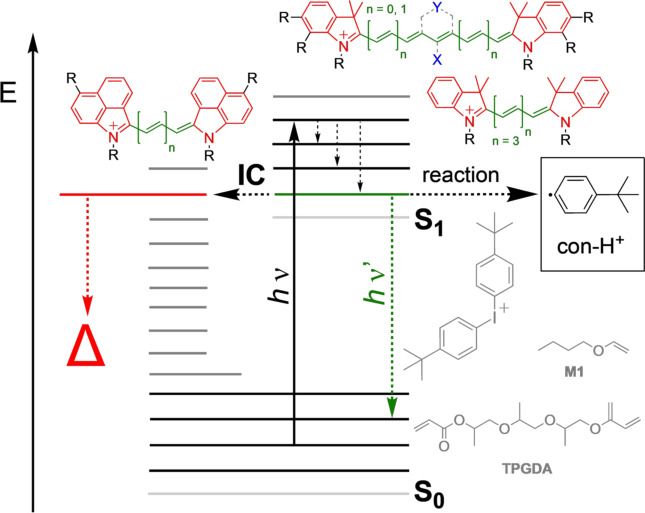

## Introduction

Cyanines[^1^, ^2^, ^3^, ^4^, ^5^, ^6^, ^7^, ^8^] comprising a heptamethine chain have received increasing attention for industrial applications based on digital recording,^[9]^ imaging,[^10^, ^11^, ^12^, ^13^] physical drying,^[14]^ chemical drying,[^5^, ^6^, ^15^, ^16^, ^17^, ^18^, ^19^, ^20^, ^21^, ^22^, ^23^, ^24^, ^25^, ^26^, ^27^, ^28^] laser marking for plastics,[^29^, ^30^, ^31^, ^32^] or laser welding.[^33^, ^34^, ^35^, ^36^] These cyanines work well as absorbers in combination with NIR lasers in industrial applications[^5^, ^6^, ^13^, ^18^] requesting the necessity that absorption of the cyanine and emission of the NIR‐source complement each other. This requires therefore also a tailoring of absorption of the conjugated system to meet these requirements. This can be achieved by change of the terminal indolium moiety; that is either an indolium‐, benzo[*e*]‐, benzo[*g*]‐, or benzo[*cd*] pattern. Available absorbers possess a variable absorption between 750 and 1100 nm where modular semiconductor devices such as lasers have been available. The reason for the preference of heptamethines can be seen in their synthetic accessibility where reaction between the respective indolium salt and the easy availability of the chain builder results in the desired cyanine structure covering its absorption in the NIR, see below.[^5^, ^6^] Additional variation of the substituent in the *meso*‐position results in fine tuning of the absorption by introduction of an electron‐donating or electron‐withdrawing group.[^1^, ^2^, ^4^, ^5^, ^7^] In addition, exchange of the hydrogen in the *p*‐position of the indolium moiety by either electron‐donating (OR, SR) or ‐withdrawing (Cl, Br, SO_3_^−^) groups also affects absorption. Here, electron‐donating substituents typically result in a bathochromic shift of absorption while electron‐withdrawing groups lead to a significant hypsochromic shift of absorbance.

In recent years, fundamental progress has been made in this field particularly to dry coatings.[^14^, ^15^, ^16^, ^17^, ^18^, ^20^, ^22^, ^23^, ^32^] This can physically occur to remove water as solvent resulting in formation of a solidified film.[^14^, ^32^] In addition, chemical drying results in crosslinked films applying either liquid monomers[^15^, ^16^, ^17^, ^18^, ^20^, ^22^, ^23^] or reactive powders.[^15^, ^17^, ^19^, ^21^] Application of the latter belongs to green technologies as well. Consequently, NIR‐lasers with line‐shaped focus^[18]^ or recently introduced high‐power NIR‐LED devices[^16^, ^32^, ^37^, ^38^] have moved into the focus of the aforementioned applications. In addition, chemical drying based on activated photoinduced electron transfer (**PET**) mostly results in colored products,[^5^, ^22^, ^37^, ^38^] which can be beneficial to design materials with readout function at certain wavelengths.

Alternatively, up‐conversion nanoparticles have received additional attention to initiate photopolymerization either in the UV or blue region.[^18^, ^39^, ^40^, ^41^, ^42^, ^43^, ^44^, ^45^, ^46^] Here, absorption of the laser light proceeds at 980 nm while a three‐ and four‐photon absorption results in formation of blue and UV light.[^40^, ^41^] This was applied to initiate either free‐radical[^40^, ^41^, ^42^, ^44^, ^45^, ^47^] or controlled radical^[41]^ polymerization. In addition, these systems can generate conjugate acid^[39]^ to initiate cationic polymerization.^[43]^ Additional interest relating to applications focused on establishing deep cure length (more than 13 cm),^[44]^ and 3D printing^[47]^ that represents some interesting features of these materials. Such NIR responsive materials generate only circa 1 % up‐converted radiation^[48]^ that is used in photonic events while the remaining part belongs to thermal energy released in the surrounding matrix.

Furthermore, there exists an approach combining physical and chemical drying of aqueous dispersions comprising crosslinkable monomers for radical polymerization.^[14]^ Here, a combination of physical NIR‐drying applying high‐power NIR‐LEDs emitting either at 820 nm, 860 nm, or 930 nm facilitated film formation of the aqueous poly(urethane) dispersion while subsequent exposure with a UV‐LED emitting at 395 nm resulted in formation of a semi‐interpenetrating polymer network by radical crosslinking of the multi‐functional acrylate monomer.^[14]^ Ethyl(2,4,6‐trimethylbenzoyl) phenylphosphinate generated radicals to initiate free‐radical polymerization of multi‐functional monomers such as tripropylene glycol diacrylate (TPGDA). This procedure resulted in films appearing colorless by eye, which can be seen as a big effort in this field.^[18]^ Herein, cyanines also served as absorber for physical drying.[^14^, ^32^]

Big efforts have been made in both radical[^5^, ^16^, ^37^, ^38^, ^49^] and cationic[^5^, ^16^, ^37^, ^49^, ^50^] polymerization using NIR‐LEDs. Photochemical generation of initiating radicals and conjugate acid based on activated **PET** explain the use of strong emissive sources such as NIR lasers[^18^, ^40^, ^41^, ^44^, ^47^] or more sophisticated high‐power NIR‐LEDs.[^5^, ^16^, ^37^, ^38^, ^49^, ^50^] Heptamethines comprising either an indolium or benzoindolium moiety fit well in this scheme.^[5]^ Particular cyanines with a dimethylene bridge in the center of the cyanine showed promising performance regarding sensitization of cationic polymerization in the NIR.[^16^, ^37^] However, those with trimethylene bridge failed to cure monomers where polymer formation proceeded by a carbo cation. This was an epoxy monomer.^[20]^

There are still open questions regarding the optimal pattern of the cyanine sensitizer in activated **PET** reacting with an initiator such as an iodonium salt.[^22^, ^51^] The sensitizer can be a conjugated system with either open or bridged polymethine chain. The pattern of the indolium moiety differs or the length of the polymethine chain can differ. Each extension of the polymethine by two methine groups typically results in a bathochromic absorption shift of 100 nm.[^1^, ^2^, ^3^, ^4^, ^5^, ^7^, ^52^, ^53^, ^54^, ^55^] In addition, comparison of a cyanine comprising an indolium moiety with that of a benzo[*cd*]indolium end group shows a 200 nm bathochromic shift of absorption.^[5]^

Nevertheless, only some of these cyanines worked well to sensitize both radical and cationic photopolymerization. There still exists a lack regarding the choice of the general cyanine pattern relating to photochemistry and photophysics in such systems. Structural features such as polymethine length, substitution of polymethine chain with bridging moiety, substitution of the *meso*‐position, and type of indolium pattern have become some targets of research. Nevertheless, focus shall be given on those sensitizers facilitating the use of high‐power NIR‐LEDs emitting between 800 and 950 nm. Nowadays, semiconductors have been available for acceptable economic conditions emitting at 860 nm or 930 nm. Thus, this contribution is going to bring more impetus in this field regarding the optimal structural pattern of the NIR sensitizer for this purpose and their efficiency to initiate radical and cationic photopolymerization.

## Results and Discussion

### Structural Selection of the Cyanine Patterns

**Scheme 1:**
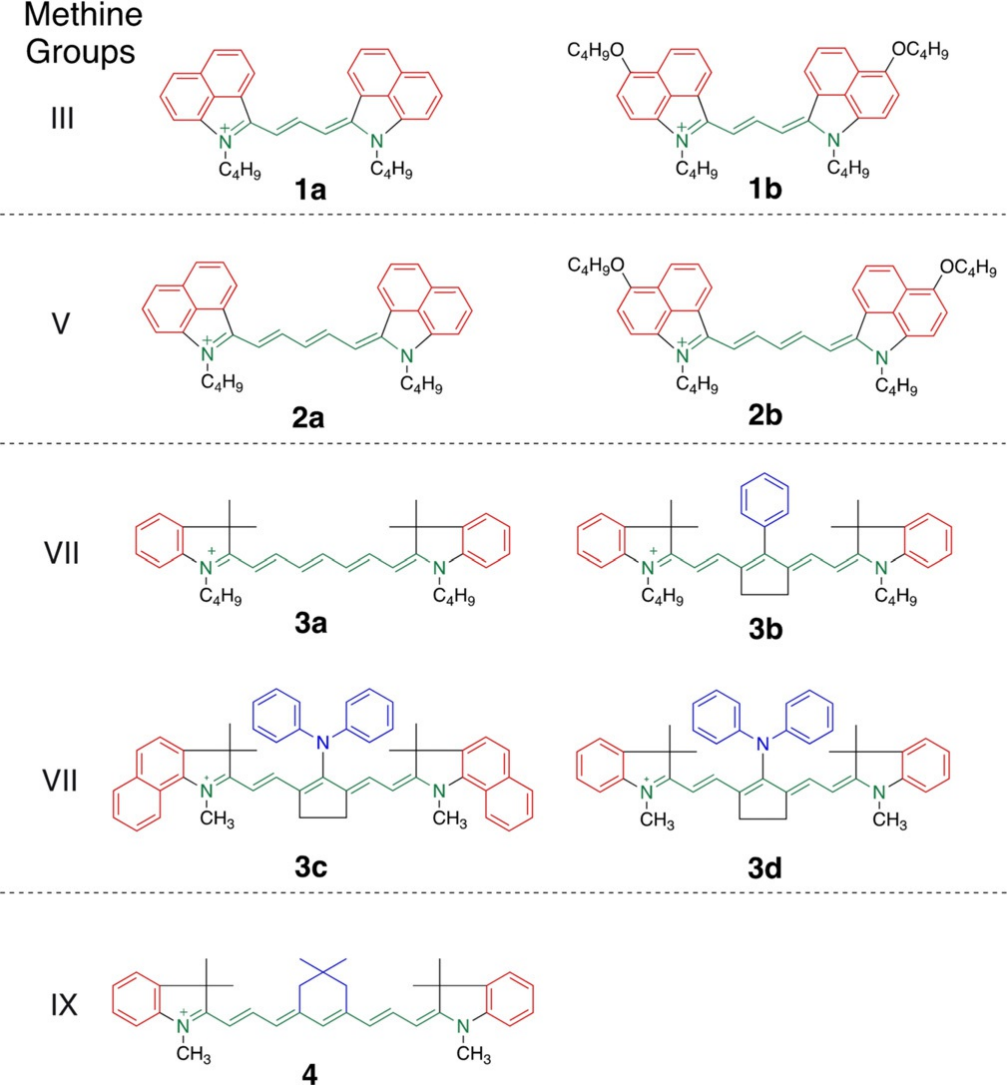
Structural pattern of the cyanine cations used to sensitize formation of conjugate acid and initiating radicals for free radical and cationic polymerization. These cations comprise the following anions: **1 a**, **1 b**, **2 a**, **3 c**, **4**: [PF_3_(C_2_F_5_)_3_]^−^; **2 b**, **3 a**: [PF_6_]^−^; **3 b**: *p*‐CH_3_‐Ph‐SO_3_^−^; **3 d**: BF_4_^−^.

Scheme 1 shows the distinct cyanines **1**–**4**. They comprise comparable numbers of π‐electrons but distinct substitution patterns. This causes different optical properties and unexpected chemical reactivity with the iodonium salt **5**, see below. Their polymethine chain comprises three (**1**), five (**2**), seven (**3**), and nine (**4**) methine groups. They were chosen as NIR sensitizers in combination with an iodonium salt to generate radicals and conjugate acid to initiate radical and cationic polymerization, respectively. A different number of methine groups connects the terminal indolium moieties resulting in a red shift of absorption, Table 1. Data shown also exhibit the expected 100 nm bathochromic shift of cyanines by extension of the polymethine chain with two additional methine groups as long as the pattern of the terminal indolium group does not change.[^1^, ^2^, ^5^] This follows by comparison data of **1 a** and **2 a** as well as **1 b** and **2 b**. Change of the benzo[*cd*]indolium pattern by an indolium group exhibits hypsochromic absorption shift. Here, absorption appears 200 nm to shorter wavelengths. Comparison between **1 a** and 1‐Ethyl‐2‐[3‐(1‐ethyl‐3,3‐dimethyl‐1,3‐dihydroindol‐2‐ylidene)propenyl]‐3,3‐di‐methyl‐3*H*‐indolium iodide (CAS 14696‐39‐0; *λ*_max_ (MeOH)=546 nm^[56]^) brought experimental approval. This also occurred if **2 a** was compared with 1,3,3‐Trimethyl‐2‐[5‐(1,3,3‐trimethyl‐1,3‐dihydroindol‐2‐ylidene)penta‐1,3‐dienyl]‐3*H*‐indolium tetrafluoroborate (CAS 38575‐74‐5, *λ*_max_ (MeOH)=638 nm^[56]^).

**Table 1:** Comparison of the photophysical data such as absorption maximum *λ*_max_, extinction coefficient *ϵ*_max_, fluorescence emission maximum *λ* ^f^_max_, fluorescence quantum yield *Φ*_f_, fluorescence decay time *τ*_f_, and rate constant for fluorescence *k*_f_ for the cyanines **1**–**4** and their respective oxidation products **3 b**–**ox**. Electrochemical data relate to their oxidation *E*_ox_ and reduction potentials *E*_red_. In addition, data also show the concentration of conjugate acid ([*a* ${}_{H{}^{+}}$ ]) formed after exposure of the system comprising the sensitizer and **5** after 15 min (see ref. [20] for more details regarding quantification of conjugate acid formed using Rhodamine B lactone, SI shows more details). The quantity *k*_b_(rel) discloses the change of sensitizer concentration after 2 min NIR exposure at either 820 nm (**1 a**, **3 a**, **3 b**, **3 d**) or 860 nm (**1 b**, **2**, **3 c**, **4**) with high‐intensity NIR‐LED device emitting with an intensity of 1 W cm^−2^. The quantity was corrected by the absorption of the sensitizer; that is, dividing of Δ[**Sens**]/Δ*t* by (1–10^−OD[Sens]^).

	*λ*_max_ [nm]^[a]^	*ϵ*_max_ [M^−1^×cm^−1^]^[a]^	*λ* ^f^_max_ [nm])^[b]^	*Φ*_f_^[b]^	*τ*_f_ [ps])^[b]^	*k*_f_ [10^9^ s^−1^]	*E*_ox_ [V]^[a]^	*E*_red_ [V]^[a]^	[*a* ${}_{H{}^{+}}$ ] [M]^[a]^	[*a* ${}_{H{}^{+}}$ ]/[**Sens**]	*k*_b_(rel) [M×s^−1^]^[a,c]^
**1 a**	758	1.28×10^5^	782	0.0031	49	0.016	0.99	−0.36	0.46×10^−6^	0.03	16
**1 b**	817	1.60×10^5^	843^[d]^	0.0011^[d]^	99	0.011	0.78	−0.51	0.45×10^−6^	0.022	11
**2 a**	859	3.23×10^5^	879^[d]^	0.0008^[d]^	<25^[e]^	n.d.^[e]^	0.80	−0.32	0.44×10^−6^	0.022	8
**2 b**	917	1.12×10^5^	^[f]^	^[f]^	^[e,f]^		0.57	−0.48	0.41×10^−6^	0.020	6
**3 a**	747	2.37×10^5^	774	0.26	1154	0.23	0.56	−0.63	5.35×10^−6^	0.3	330
**3 b**	786	3.23×10^5^	824	0.19	1008	0.19	0.58	−0.56	1.17×10^−6^	0.06	416
**3 b‐ox**	677	2.29×10^5^	708	0.31	1313	0.24	0.58	−0.56	2.05×10^−6[g]^		
**3 c**	835	2.11×10^5^	869^[d]^	0.0076^[d]^	213	0.034	0.54	−0.61	3.87×10^−6^	0.19	425
**3 d**	794	2.47×10^5^	835	0.066	934	0.071	0.57^[16]^	−0.60^[16]^	2.05×10^−6^	0.11	97
**4**	854	1.83×10^5^	884^[d]^	0.0149^[d]^	285	0.052	0.46	−0.33	2.01×10^−6^	0.2	1000

[a] In acetonitrile. [b] In ethanol. [c] *k*_b_: [**Sens**]:[**5**]=1:6 (molar); **5**=3.0×10^−5^ mol L^−1^. (One should keep in mind that the emission of the LED is not tunable. Therefore, excitation at the edges or the maximum results in different penetration length taking similar conditions of the sensitizers.) [d] Determined with respect to a reference (Sulforhodamine 101)^[57]^ using a Lambda 1050 WB (PerkinElmer) with spectral extension to access the stationary fluorescence data. [e] Below the detection limit of the instrument; that is, ≥25 ps. [f] N.d. due to tiny emission efficiency recorded with a Lambda 1050 WB (PerkinElmer) with extended spectral shift (up to 1100 nm). [g] Determined by exposure with a 820 nm NIR‐LED (I=1 W cm^−2^), there was a remarkable reactivity although absorption contributes only little at this wavelength.


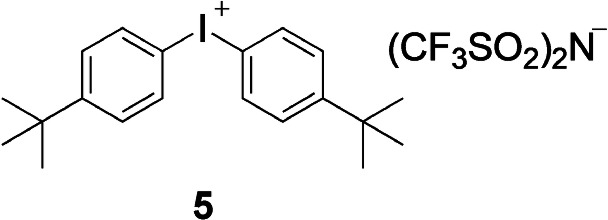


Substitution of the 4‐position with respect to the indolium nitrogen in **1 a** and **2 a** by an alkoxy group results in **1 b** and **2 b**, respectively. This leads to 60 nm bathochromic absorption shift. Change of the benzo[*cd*]indolium moiety in **2 a** by indolium and increase by additional two methine groups in the connecting polymethine chain results in **3 a** exhibiting a hypsochromic absorption shift of about 100 nm. **3 a** assigns to a heptamethine while additional extension of the polymethine results in the nonamethine **4** exhibiting a bathochromic shift of about 100 nm.

The absorption of the heptamethine pattern of **3 b**–**3 d** overlaps with the emission of the 860 nm NIR‐LED either at the absorption tail of **Sens** (**3 b**, **3 d**) or the absorption maximum of **Sens** (**3 c**). A dimethylene bridge in the center of the conjugated system keeps the geometry nearly planar.^[38]^ Similar structural patterns of **3 b** and **3 d** contributed to initiation of radical and cationic polymerization[^5^, ^16^, ^49^] while heptamethine‐based cyanines with a moiety comprising a trimethylene bridge in the center failed regarding initiation of cationic polymerization.^[20]^ Formation of nucleophilic photoproducts inhibits cationic polymerization. In addition, **4** assigns to a nonamethine cyanine in which the substituted trimethylene bridge slightly distorts the planarity of the conjugated chain. **3 a** comprising no bridging elements at the polymethine showed an almost planar geometry in quantum chemical calculations. Figures SI7–13 depict the geometries of **1**–**4**. Bridging of the central position with two methylene groups results in **3 b** and **3 c** exhibiting almost a flat pattern of the NIR‐absorber. Absorber **4** also exhibited planarity.

Electrochemical experiments complement the properties of the conjugated structures. The oxidation potential (*E*_ox_) drops by increase of the polymethine chain as concluded by comparing **1 a** and **1 b** as well as **2 a** and **2 b**. As expected, the introduction of an additional electron‐donating substituent results in an easier oxidation as shown by **1 b** and **2 b**. Interestingly, **2 b**, **3**, and **4** possess similar ability to oxidize although they exhibit distinct substitution patterns and number of π‐electrons. Thus, it does not really matter whether the connecting conjugated chain was open as in **2 a**/**3 a** or bridged as in **3 b**–**d**. The latter comprises either an amino or phenyl substituent in the *meso*‐position.

However, the situation changes when comparing the reduction potentials (*E*_red_) of **1**–**4**. Here, **1 a**, **2 a**, and **4** exhibit similar data although they possess different structural patterns. *E*_red_ of **3** appears at about −0.6 V demonstrating less efficient reduction capability compared the compounds, see above. Nevertheless, the systems disclosed report about generation of conjugate acid and initiating radicals generated according to an oxidative mechanism, where *E*_ox_, *E*_red_ of the initiator and the excitation energy (*E*_00_) of the sensitizer determine whether the reaction can proceed from a thermodynamic point of view. Thus, the free reaction enthalpy (Δ*G*_el_) can be written as Δ*G*_el_=*F*(*E*_ox_−*E*_red_)<M*‐>E*_00_ (*F*=Faraday constant).[^58^, ^59^] According to these data, Δ*G*_el_ appears around −0.2 eV in the case of **1 b. 1 a**, **2**, and **3 c** show Δ*G*_el_ around −0.3 eV while that of **3 b** and **3 d** appears at −0.4 eV. **3 a** and **4** exhibited the lowest Δ*G*_el_‐values with −0.5 eV and −0.7 eV, respectively. These changes can be seen more or less as moderate comparing those of **1** and **2** as well as data of **3 b**–**d**. This generally indicates that **PET** should occur from a thermodynamic point of view. The cation of **5** served as oxidizing substrate exhibiting *E*_*r*ed_ of −0.69 V.^[10]^ Solvent effects, internal reaction coordinates,^[60]^ and internal activation barriers[^16^, ^38^, ^60^] are not included here. Nevertheless, results obtained demonstrate huge differences regarding the reactivity.

### Properties of the First Excited Singlet State

Stationary and time‐resolved fluorescence measurements explain the emission characteristics of the excited state to receive a deeper understanding of the S_1_ considering the NIR‐sensitizers shown in Scheme 1. Here, **1 a**, **1 b**, and **2 a** exhibit both low fluorescence quantum yield and decay time. Some of these data (**1 a** and **2 a**) reside in the range of a previous report.^[61]^ Non‐radiative processes mainly contribute to the overall behavior of the excited state, Table 1. Size increase of the conjugated pattern decreases excitation energy resulting in an increase of internal conversion (IC). Obviously, there exists in these structures a higher probability where a vibration with higher energy of the S_0_ may couple with the lowest vibration of the S_1_ favoring IC.^[62]^ Those non‐radiative events therefore proceed more efficient in the case of absorbers comprising a benzo[*cd*]indolium pattern such as **1** and **2** as shown by the low *τ*_f_ and *Φ*_f_ data, Table 1. This follows by comparison with **3** and **4** exhibiting higher values, and of course higher reactivity with **5** as documented by the bleaching constant *k*_b_ and the amount on conjugate acid formed with respect to sensitizer concentration ([*a* ${}_{H{}^{+}}$ ]/[**Sens**]), Table 1. In general, non‐radiative events can lead to temperature increase in adiabatic systems, which promotes overcoming internal activation barriers in **PET** existing in similar systems.[^6^, ^16^, ^38^] In addition, the fluorescence decay time of **2 a** was lower as the time resolution of the single photon equipment used to study dynamics of the excited state; that is, >25 ps. Time‐resolved data were not available in the case of **2 b** because spectral response of our instrument ends above 910 nm.

In addition, increase of the conjugated system from **1 a** to **2 a** by insertion of an additional CH=CH group as expected results in a decrease of the energy gap between the ground state and the excited state. This therefore leads to a higher probability of energetically higher available vibrations of the ground state (S_0_). It favors internal conversion (IC) between the excited singlet state (S_1_) and S_0_. Here, only total‐symmetric vibrations couple with the symmetry‐allowed electronic transition.^[63]^

Excess of vibrational energy is distributed by vibrational relaxation (VR) to the isoenergetic asymmetric and symmetric states related to intramolecular vibrational redistribution (IVR).^[64]^ The electronic spectra of cyanines exhibit a vibrational fine structure where the energy difference between the sub‐bands resides at about 1200 cm^−1^ ±200 cm^−1^.[^65^, ^66^] The dominant symmetric carbon–carbon valence vibration of the polymethine chain determines spacing between sub‐bands.[^65^, ^66^] Nevertheless, the molecule still remains in the S_1_ exhibiting a lifetime in the ps and sub‐ns frame after VR of the S_1_, Table 1. Although VR within the S_1_ results in release of thermal energy, the contribution to the overall amount of heat released into the surroundings can be seen more or less as minor amount.

IC is believed to be the main source for releasing heat into the surroundings.^[5]^ Here, the energetically lowest vibration of the S_1_ (v′=0) couples with a higher vibration of the S_0_ (v=*n*) resulting in a very hot molecule. This proceeds according to the above‐mentioned prerequisites. Practically, this excess of energy is redistributed by vibrational cooling (VC).^[64]^ It transfers its excessive energy by collision with matrix molecules relating to vibrational cooling. Recent studies showed several examples where the temperature can rise higher than 100 °C and sometimes even more.[^14^, ^16^, ^17^, ^18^, ^19^, ^32^, ^37^, ^38^] In the case that no VC would proceed, the extremely hot molecule would burn because it possesses so much energy that it cannot be fully transferred to IVR.

**Scheme 2:**
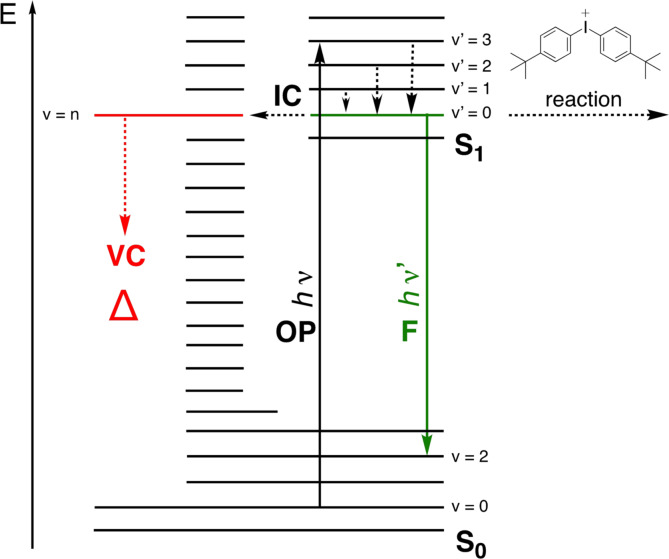
Schematic representation of the electronic ground state (S_0_) with its vibrational states v=0, v=2, and v=*n*, the first electronic excited state (S_1_) with the respective vibrational states v′=0, v′=1, and v′=2, and the vibronic (vibrational–electronic) transition where one‐photon absorption (OP) proceeds with the energy *hν*. This is followed by relaxation from the higher vibrational levels into the lowest vibrational level (v′=0) of the first excited singlet state by transfer of excess vibrational energy into isoenergetic vibrations by intramolecular vibrational energy distribution (IVR). This finally results in population of the lowest vibrational level of the S_1_ (see also ref. [64]). The S_1_ reacts either with the cation of **5** resulting in formation of initiating radicals and conjugate acid or it can couple with a higher available vibration of the S_0_ resulting in the release of heat caused by vibrational cooling (VC) as discussed earlier.^[64]^

In general, the evolution of heat in systems exhibiting large contribution of IC has not been well understood. Scheme 2 depicts the occurring processes from the simplest point of view keeping in mind that the scenario appears more difficult.[^62^, ^64^] Experiments pursued in this contribution indicated a temperature increase using TPGDA as matrix upon exposure at 860 nm, see Figure SI14. Temperature evolution observed describes the overall increase as affected by the heat capacity of the film. In general, this temperature can be much higher as, for example, observed during the burning of CD‐R and DVD‐R where cyanines also took a key function.^[9]^ It exceeded for a short moment a value above the decomposition temperature of the absorber as indicated by its bleaching. In general, the lower the excitation energy, the higher the rate of non‐radiative deactivation as shown for several emission studies of cyanines.[^5^, ^10^, ^20^]

Equation 1 shows the relation between fluorescence quantum yield *Φ*_f_, fluorescence decay time *τ*_f_, rate constant for fluorescence *k*_f_, non‐radiative deactivation *k*_nr_, and a reaction based on activated **PET** *k*_PET_. Thus, the larger the contribution of non‐radiative events, the lower become *Φ*_f_ and *τ*_f_, and therefore, also the lower the probability to participate in **PET**. Since internal conversion proceeds according to unimolecular kinetics, this reaction favors deactivation of the excited state rather than a bimolecular reaction as expressed by *k*_PET_×[Q] ([Q]=concentration of the substrate reacting with the S_1_). Fluorescence measurements cannot distinguish whether internal conversion or **PET** dominates the non‐radiative deactivation process. Here, study of photoproducts as shown below gives deeper insights into the mechanism.

(1) $$\Phi_{f}=\frac{k_{f}}{k_{f}+k_{nr}+k_{PET\times \left[Q\right]}}=k_{f}\times \tau_{f}$$

**Figure 1:**
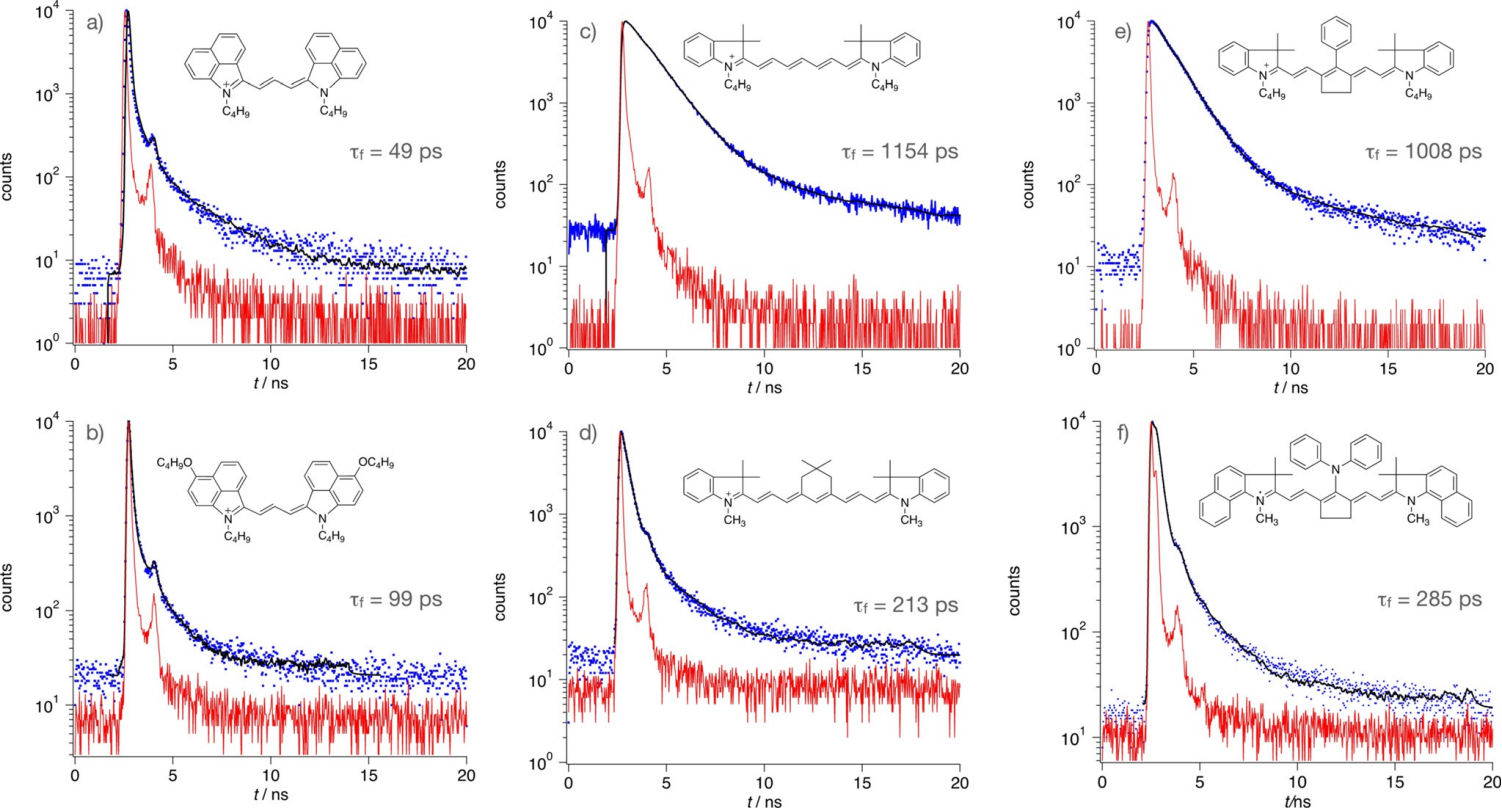
Fluorescence decay traces of the absorbers **1**–**4** in MeOH obtained after excitation at 670 nm (a: **1 a** (λ_em_=782 nm); b: **1 b** (λ_em_=843 nm); c: **3 a** (λ_em_=774 nm); d: **4** (λ_em_=884 nm); e: **3 b** (λ_em_=824 nm); f: **3 c** (λ_em_=869 nm)). SI provides more experimental details (red: instrumental response of a scatter comprising Ludox in water whose detection was close to the excitation wavelength at 670 nm, blue: decay curve of the sample, black: calculated decay by iterative convolution between the instrumental response function and exponential decay). Time‐correlated single photon counting was applied to collect the data.

Figure 1 exhibits representative fluorescence decays of some sensitizers (**1 a**, **1 b**, **3 a**–**c**, and **4**). Data obtained give access to *k*_f_, providing information how efficient radiative deactivation by fluorescence can proceed. It shows about 10 times higher values in the case of **3 a** and **3 b** compared to **1 b**. Obviously, the benzo[*cd*]indolium moiety favors non‐radiative deactivation. Theoretical considerations discuss the enhancement of this process by vibrational coupling.^[61]^ Thus, the longer polymethine chain may direct the system to more radiative deactivation while larger stiff conjugated patterns of the terminal group as available in **1** move it toward non‐radiative deactivation. Interpretation of data does not go straightforward by comparison with those of **3. 3 a** and **3 b** exhibit both similar *τ*_f_ and *Φ*_f_ resulting logically in comparable *k*_f_ although **3 a** possesses an open polymethine chain. Moreover, **3 b** comprises a bridge with two methylene groups in the center of the polymethine and a phenyl ring in the *meso*‐position.

Replacement of the phenyl group in **3 b** by a diphenyl amino group results in **3 d** showing similar fluorescence decay of 934 ps, Table 1. *Φ*_f_ dropped caused by replacement of Ph by N(Ph)_2_ in the *meso*‐position. Consequently, *k*_f_ decreased. In addition, **3 c** exhibits a lower decay rate indicating that both benzo[*g*]indolium and N(Ph)_2_ significantly contributed to the fluorescence dynamics. The *k*_f_ value of **3 c** is about a half compared to **3 d**. Moreover, an electron‐withdrawing group as the phenyl ring in **3 b** did not cause significant changes of both non‐radiative and radiative deactivation in comparison with the respective diphenyl amino compound **3 d. 3 c** possesses a lower emission decay rate. One can summarize in brief: *τ*_f_(**3 b**)=1008 ps, *τ*_f_(**3 c**)=213 ps, *τ*_f_(**3 d**)=934 ps.

Interestingly, the oxidized photoproduct, available after **PET** of **3 b** resulting in **3 b**‐**ox**, showed similar decay rates although the oxidized dimethylene bridge comprises two additional π‐electrons. Its electronic pattern appears similar to that of fulvenes.[^16^, ^38^] Thus, these structural changes in **3 b**‐**ox** do not significantly affect the decay dynamics as concluded with those of the respective cyanine **3 b**. Data also demonstrated sufficient reactivity with **5** although there was only a small overlap between absorption of **3 b**‐**ox** and **5**, Table 1.


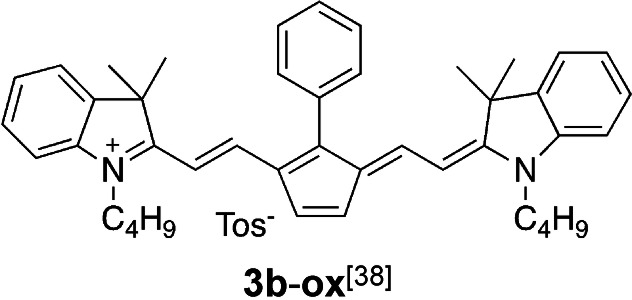


Moreover, cyanine **4** exhibiting a nonamethine chain between the indolium rings has a shorter decay time. Its *k*_f_ resides in the range of **3 c** although it comprises a longer polymethine chain between the benzoindolium rings. The significant bathochromic‐shifted absorption of **4** compared to **3** results in a decrease of the energy gap between ground and excited state therefore resulting in an increase of non‐radiative deactivation such as internal IC.

### Photochemistry and Formation of Initiating Radicals

The sensitizers **1**–**4** possess distinct structural features related to:

- absorption change as concluded by comparison of indolium (**3 a**, **3 b**, **3 d**, **4**), benzo[*g*]indolium (**3 c**), benzo[*cd*]indolium (**1**, **2**) derivatives
- open methine (**1**, **2**, **3 a**) and bridged methine (**3 b**–**d**, **4**) chains
- substituted benzo[*cd*]indolium (**1 b**, **2 b**) and those with no additional substituents (**1 a**, **2 a**)
- different bridges of the polymethine chain by variation of the substituent in the *meso*‐position, which can be either hydrogen (**4**), phenyl (**3 b**), or N(Ph)_2_ (**3 d**)
- variable indolium end groups

These structural changes affect absorption and in case of **1** and **2** also the redox potential as shown in Table 1, see above.

**Figure 2:**
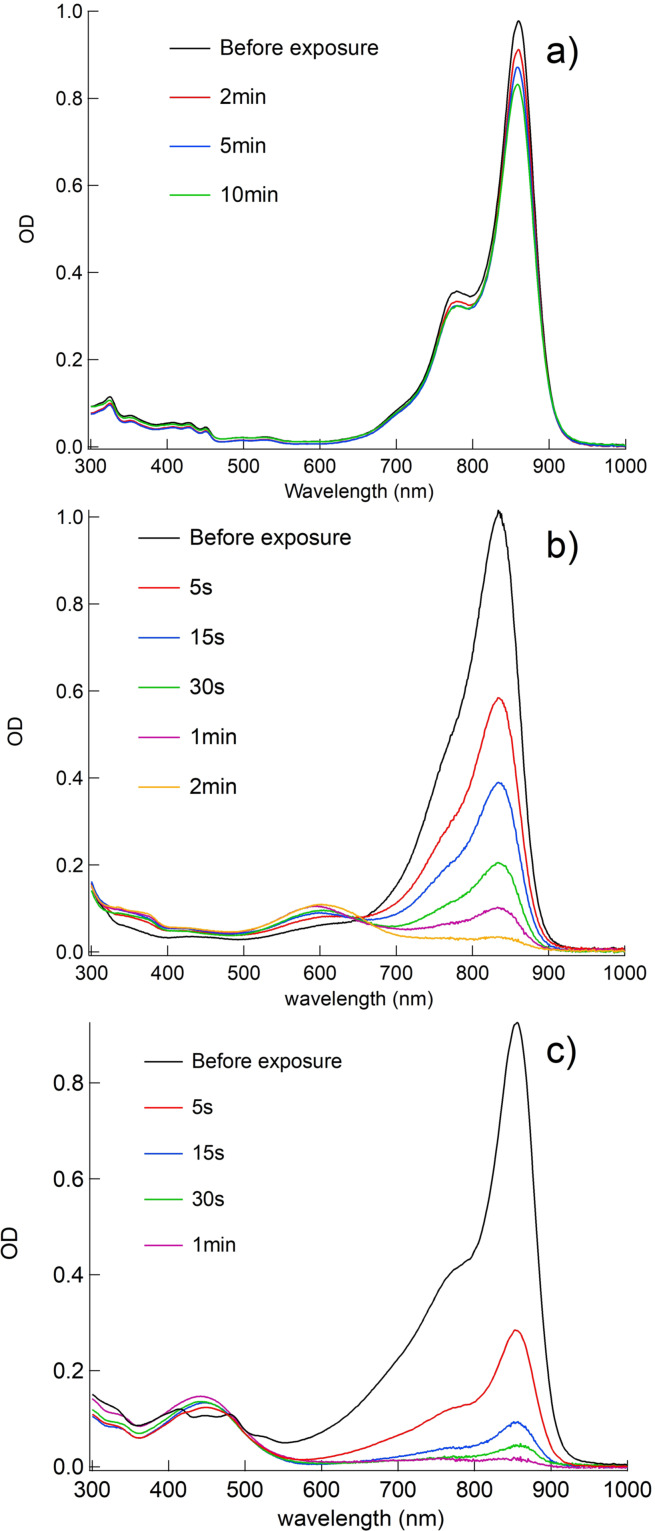
Decrease of OD upon exposure of NIR‐sensitizer in the presence of **5** measured in acetonitrile using a NIR LED emitting at 860 nm with an intensity of 1 W cm^−2^; a) **2 a**, b) **3 c**, c) **4** ([**Sens**]/[**5**] =1/6).

Consideration of Δ*G*_el_ by using the oxidation potential of the sensitizers and the reduction potential of **5** (*E*_red_=−0.69 V^[10]^) results in slight negative values of Δ*G*_el_. Thus, **1**–**4** should exhibit similar reactivity since Δ*G*_el_ appears similar. However, data obtained for bleaching (*k*_b_) and generation of conjugate acid (*a* ${}_{H{}^{+}}$ ) shown in Table 1 require a discussion of a scenario that requires inclusion of additional points to understand these reactivity differences. These data represent the chemical reactivity of the system. Figure 2 shows the spectral changes obtained upon exposure of **Sens** in the presence of **5** with an 860 nm high‐intensity LED. This includes a benzo[*cd*]indolium derivative (**2 a**) with open polymethine chain (5 methine groups) in Figure 2 a, benzo[*g*]indolium derivative (**3 c**) with dimethylene bridged polymethine chain (7 methine groups) in Figure 2 b, and an indolium derivative (**4**) with trimethylene‐bridged polymethine chain (9 methine groups) in Figure 2 c.

Data obtained for the decrease of sensitizer absorption in combination with **5** during exposure indicate either a slow decrease of OD (Figure 2 a), a significantly faster decrease and therefore also bleaching at the exposure wavelength (Figure 2 b), and finally the fastest decrease of OD with formation of a new absorption band between 400 and 500 nm (Figure 2 c). General structures **1** and **2** are related to the small spectral changes shown in Figure 2 a while **4** is assigned to Figure 2 c. **3 c** resulted in the pattern shown in Figure 2 b where a new absorption appears hypsochromically shifted at 600 nm. This may relate to the oxidized species exhibiting a fulvene pattern as similarly disclosed in the case of **3 b**‐**ox**.^[16]^ These different bleaching efficiencies enable to draw the following reactivity ratio; that is, according to Table 1 *k*_b_(rel):

*k*_b_(rel): **4**>**3 c**≈**3 b**≳**3 a**>**3 d**≫**1 a**≈**1 b**≈**2 a**≈**2 b**

It should also result in a similar ratio considering the formation of conjugate acid [*a* ${}_{H{}^{+}}$ ].^[20]^ Photoinduced electron transfer between photoexcited **Sens** (Sens*) and **5** results in the oxidized form **Sens^+.^**, Equation 2, which stabilizes by release of conjugate acid [*a* ${}_{H{}^{+}}$ ] and photoproducts comprising nitrogen (Pr(N)) and those with no nitrogen (Pr(O)), Equation 3. This equation shows that one mole of conjugate acid should relate to one mole sensitizer. Photoproducts comprising amino groups (Pr(N)) can additionally react with **Sens^+.^** that yields back **Sens**, Equation 4. In addition, protonation of Pr(N) according to Equation 5 also reduces the available amount on conjugate acid needed to protonate colorless Rhodamine B lactone resulting in deep red colored Rhodamine B. This reaction was qualified to probe quantitatively the amount on acid formed.^[20]^ It can additionally explain why its available amount can be smaller than expected.

(2) $$Sens{}^{*}+5\to Sens{}^{+}{}^{•}+5{}^{-}$$

(3) $$Sens{}^{+}{}^{•}\to a{}_{H{}^{+}}+\mathrm{Pr}\left(N\right)+\mathrm{Pr}\left(O\right)$$

(4) $$Sens{}^{+}{}^{•}+\mathrm{Pr}\left(N\right)\to Sens+\mathrm{Pr}\left(N\right){}^{+}$$

(5) $$a{}_{H{}^{+}}+\mathrm{Pr}\left(N\right)\to \mathrm{Pr}\left(N\right)\text{-}a{}_{H{}^{+}}$$

According to the data shown in Table 1, the following ratio was obtained for the formation of conjugate acid upon exposure of **Sens** in the presence of **5**.

[*a* ${}_{H{}^{+}}$ ]/[**Sens**]: **3 a**>**3 c**>**4**≈**3 d**>**3 b**≫**1 b**≈**2 a**≈**2 b**≈**1 a**

Both *k*_b_(rel) and [*a* ${}_{H{}^{+}}$ ]/[**Sens**] demonstrate the higher reactivity of derivatives comprising longer polymethine chains (**3 a**, **4**) while those with stiff benzo[*cd*]pattern (**1 b**, **2 a**, **2 b, 1 a**) showed less reactivity. Thus, **3** and **4** result in higher reactivity with **5**. Particularly, **4** exhibits the most promising features as **Sens** in NIR‐sensitized photopolymerization in combination with more sophisticated 860 nm high‐power NIR LED devices. It demonstrates high reactivity based on data for bleaching.

**Scheme 3:**
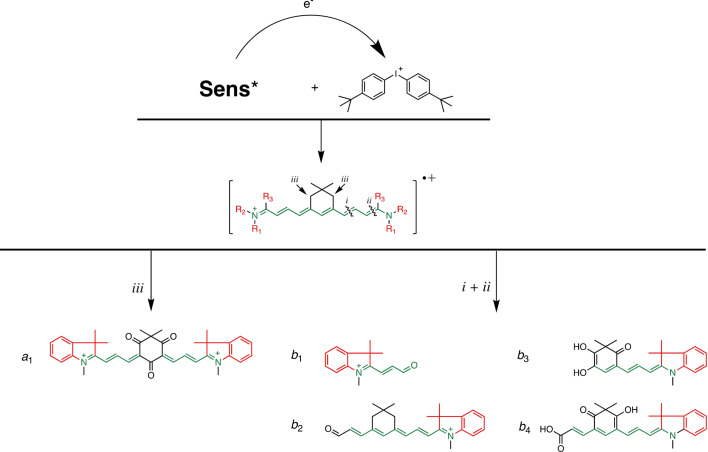
Proposal of structural patterns based on the mass of the molecular ions de detected by LC–MS spectrometric experiments by analysis of the molecular ions available in their mass spectra upon exposure of **4** in the presence of **5** at 860 in CH_3_CN (More experimental details in SI).

Mass spectrometric analysis obtained by a LC–MS analytical protocol enabled to draw possible pathways based on the molecular ions detected as shown in Scheme 3 in the case of **4** (for details see SI). Explorative MS studies of exposed solutions comprising **3 b** and **3 d** were previously reported.[^16^, ^38^] Thus, **3 c** should exhibit similar behavior compared to **3 c**. The reaction between Sens* and **5** resulted either in oxidation of position *iii* or bond cleavage at the position *i* and *ii* resulting in formation of nucleophilic products. For simplification, sensitizers exhibiting an open chain such as **1**, **2**, and **3 a** were not included here since molecular ions observed exhibited a mass related to cleavage of the adjacent bond with respect to the indolium moiety. Surprisingly, **4** showed oxidation activity at its *iii*‐position resulting in yellowish photoproducts as shown in Figure 2 c while the substrate appeared optically open at the excitation wavelength. Such unexpected behavior may facilitate the design of photoswitching systems. Basically cleavage at either the *i* or *ii* position was expected as main pathway since heptamethine derivatives with similar trimethylene bridge in the center showed this behavior with no indication of products based on oxidation of position *iii*.^[22]^ Scheme 3 summarizes the reaction products observed in case of **4** as a proposal derived from the molecular ions found in the MS spectra. The formation of this product mixture may also explain why absorption spectra in Figure 2 c do not go through an isosbestic point which typically occurs in case of photochemistry proceeding from A*→B.^[67]^ Structures *b*_2–4_ may explain the hypsochromic‐shifted absorption whose pattern does not belong anymore to a cyanine.

### Activated Free‐Radical Polymerization

Recent investigation of cyanines as NIR‐sensitizers showed the necessity to introduce additional heat needed for **PET** with diaryliodonium salt exposed at 805 nm.[^16^, ^38^] An internal activation barrier requires the introduction of additional heat to succeed with **PET** in the case of sensitzers comprising cyanine patterns.[^6^, ^16^, ^37^, ^38^] This can optimally occur under adiabatic conditions with a sensitizer exhibiting a large contribution of non‐radiative deactivation, Table 1. Only sensitizers comprising a barbiturate and therefore no charge required significant lower exposure intensity to initiate successful radical photopolymerization of the monomer TPGDA.[^10^, ^20^, ^22^, ^51^] In addition, no successful result has been reported regarding the use of a cyanine sensitizer exhibiting an open methine chain between the terminal indolium groups where excitation above 750 nm resulted in generation of aryl radicals and conjugated acid applying **5** as coinitiator. Sensitizers used in preliminary studies comprised a connecting bridge.[^16^, ^18^, ^22^]

There does not exist a clear answer how the structural pattern of the cyanine‐based sensitizer affects the internal barrier of the **PET**. This relates to the length of the polymethine chain and its sensitizing efficiency in **PET** applying either high or low exposure intensity in the NIR. There exist a few reports about successful application of cationic cyanines but research did not go into much detail with respect to the structure.[^16^, ^38^] Thus, comparison of **1**–**4** may give some answers showing whether an open chain as in **1**, **2**, and **3 a** or those comprising an aliphatic bridge as depicted in the case of **3 b**–**3 d** and **4** affect the sensitization efficiency. In general, it should follow the reactivity as shown by *k*_b_ in Table 1 applying a higher‐intensity NIR‐LED.

In addition, the different pattern of the indolium moiety might affect sensitization efficiency as concluded by comparison between those comprising benzo[*cd*]indolium (**1**, **2**), benzo[*g*]indolium (**3 c**), and indolium pattern (**3 a**, **3 b**, **3 d**, **4**). In particular, these structures might also affect the size of the internal activation barrier of **PET**. The tendency of stronger non‐radiative deactivation of **1** and **2** may move them into the focus since a combination of sensitizer and **5** also resulted in a decomposition temperature below 100 °C.^[20]^ According to the reactivity above‐mentioned results, structures **3** and **4** should lead to higher reactive systems, while **1** and **2** would not follow.

Preliminary studies indicated the possibility to overcome internal activation barrier of systems comprising **3 b** and **3 c** in combination with the cation of **5**[^16^, ^38^] and a high‐intensity NIR‐LED. Structures **1**, **2**, **3 a**, and **4** appear new in this application field. It was also shown that a dimethylene bridge as shown in **3 b** and **3 d** favors cationic polymerization,[^16^, ^37^, ^38^] while sensitizers with trimethylene bridge failed.^[20]^ Surprisingly, **3 a** exhibited acceptable radical photopolymerization upon exposure with low‐intensity emitting NIR‐LEDs; that is, 45 mW cm^−2^. None of **1**, **2 a**, **3 b**‐**c**, or **4** sensitively responded at this low intensity. Exposure in a photo‐DSC setup applying higher intensity (*λ*=860 nm, *I*=350 mW cm^−2^) gave similar results. Here, the experimental conditions in the photo‐DSC facilitate almost isothermal conditions. Thus, heat released by the sensitizer and also by polymerization would not be accessible by the system to overcome internal activation barriers. However, this differs in the case of a real‐time FTIR setup where heat formed in the reaction does not efficiently leave the system, rather resulting in conditions that reside between an adiabatic and isothermal system in the time frame of the reaction. This has often facilitated NIR‐sensitized radical photopolymerization needing both photons and heat to proceed **PET** according to an activated scheme.[^5^, ^16^, ^37^, ^38^]

**Figure 3:**
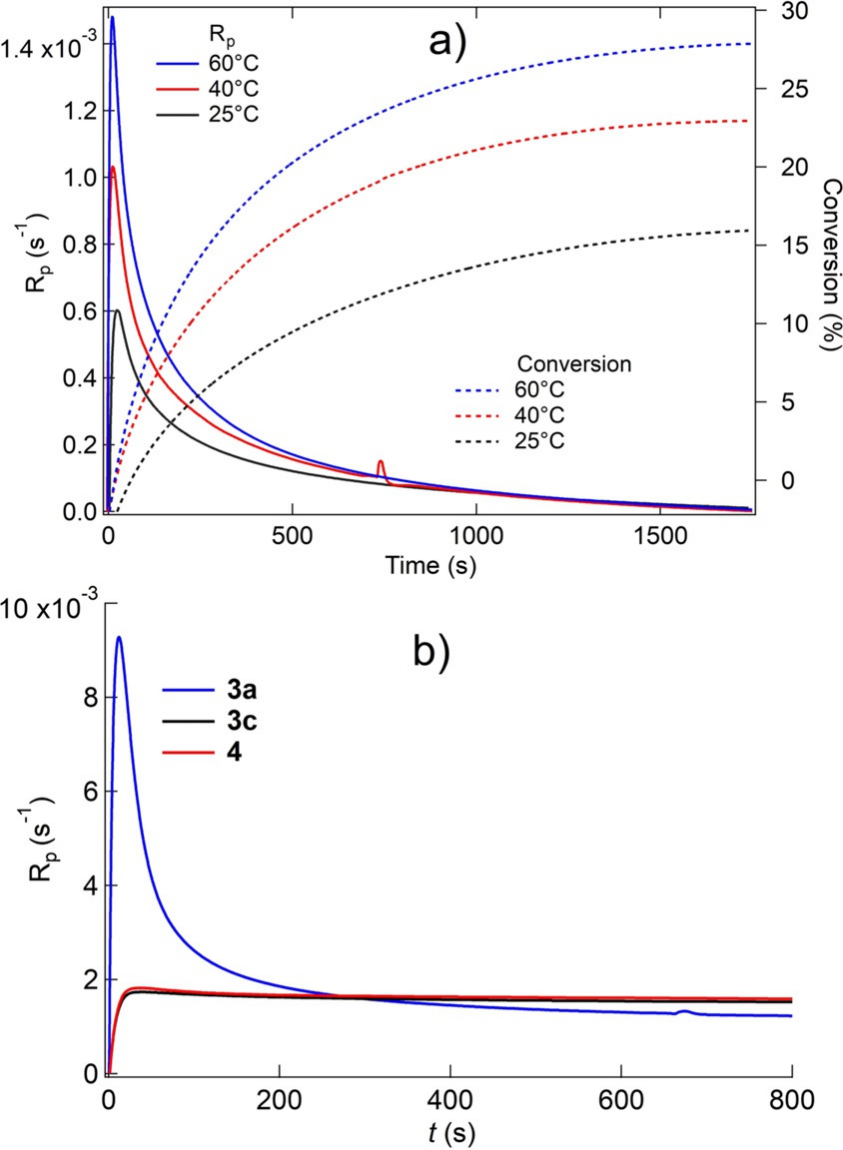
Conversion–time and polymerization rate (*R*_p_) profiles of the monomer TPGDA obtained with the initiator system comprising NIR sensitizer and the initiator **5** (1.0 wt %, [**Sens**]:**5**=1:6 (molar)) exposed at 770 nm (*I*=45 mW cm^−2^) measured by photo‐DSC taken at different temperatures (Figure 3 a; **Sens**=**3 a**) as shown in Figure 3 b at 40 °C with different sensitizers exhibiting either an open polymethine chain (**3 a**) or bridged polymethine chain (**3 c**, **4**) exposed at 820 nm (*I*=200 mW cm^−2^) for **3 a** and at 860 nm (*I*=350 mW cm^−2^) for **3 c** and **4**.

Surprisingly, **3 a** exhibited an acceptable photopolymerization efficiency using a low intensity LED (*λ*=770 nm, *I*=45 mW cm^−2^), Figure 3 a. On the other hand, the remaining cationic sensitizers **3 c** and **4** did not show remarkable polymerization. Temperature increase in the DSC did not change the scenario. Only **3 d** slightly responded.^[16]^ Thus, **3 a** can be seen as an interesting alternative compared to **3 b**–**d** in combination with low‐intensity NIR‐LEDs because it exhibits a lower intrinsic activation barrier as concluded by the higher reactivity, Figure 3 a,b. Obviously, cyanines with unbridged polymethine chain result in **PET** with **5** even under low exposure conditions if they bear an indolium moiety as terminal group. As a consequence, the threshold to initiate radical polymerization is lower in **3 a**. This might also open new perspectives for applications operating with significantly lower intensity.

### Mediated Acid‐Initiated Cationic Polymerization Self‐Polyaddition of 4‐(Hydroxybutyl) Vinyl Ether

**Figure 4:**
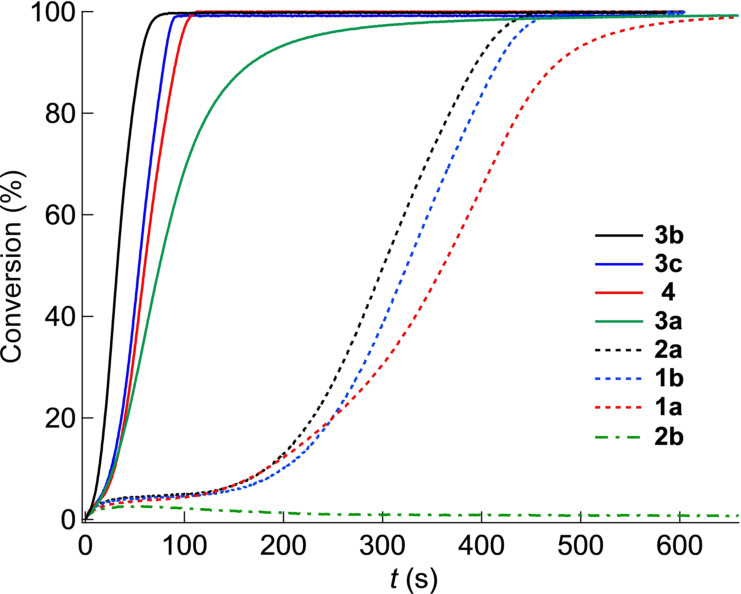
Conversion–time profiles of the vinyl ether **M1** obtained with the initiator system comprising initiator **5** (2.0 wt %) and NIR sensitizer ([**Sens**]:**5**=1:6 (molar ratio)) exposed at 860 nm (*I*=1.0 W cm^−2^) in the case of **4**, **3 c**, **2**, and **1 b** and 820 nm (*I*=1.0 W cm^−2^) in the case of **3 a**, **3 b**, and **1 a** measured by real‐time FTIR.


Table 1 shows substantial generation of conjugate acid in the case of **4** and **3 c** while it results in less quantity in the case of **1**, **2**, and **3 b**. Nevertheless, **3 a** generated the largest amount on conjugate acid. Figure 4 shows these reactivity differences, which differently appear in the reactivity of the monomer 4‐(hydroxybutyl) vinyl ether (**M1**). In previous investigations this monomer exhibited fast reactivity^[68]^ with sensitizers comprising indolium terminal groups in combination with an iodonium salt derived from aluminates ([Al(O‐*t*‐C_4_F_9_)_4_]^−^).^[37]^

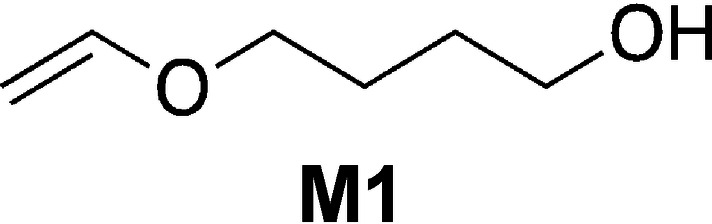
 
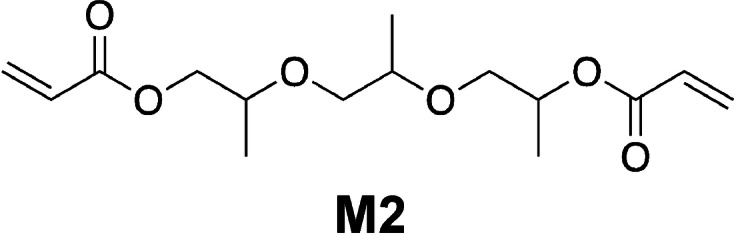


Figure 4 clearly demonstrates the fast reactivity of the bridged derivatives **3 c** and **4** in combination with **5** whose quantitative amount of conjugate acid remains at significantly higher level compared to **2 a** and **1 b. 3 b** also reacted fast while **3 a** appeared less reactive compared to the above‐mentioned sensitizers of group **3**. Here, bond cleavage of the polymethine chain results in formation of nucleophilic products inhibiting cationic photopolymerization. Thus, oxidation of position *iii* favors formation of less nucleophilic products in the case of **3 b**–**d** explaining the higher reactivity shown in Figure 4. In this series, **3 a** possesses a higher reactivity compared to **1** and **2**. Thus, the introduction of sensitizers exhibiting a benzo[*cd*]indolium pattern has not brought progress in this field since these cyanines mostly favor non‐radiative deactivation rather than to react with **5** by **PET**. It appears more likely that incorporation of a longer methine chain as in either **4** or **3 c** favors the reactivity in NIR‐sensitized acidic catalyzed polymer formation of **M1**. It also shows that these systems worked with **5** comprising an anion that typically failed in cationic photopolymerization; that is, [(CF_3_SO_2_)_2_N]^−^.^[20]^

**Scheme 4:**
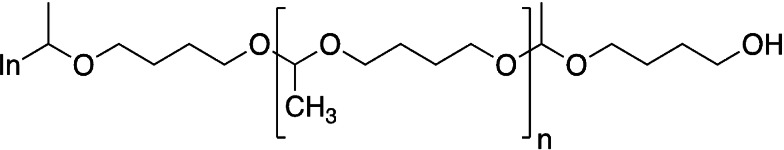
Structural pattern of the product formed by acid‐catalyzed self‐polyaddition according to refs. [70, 71].

The molecular weight of the isolated polymer indicated a *M*_n_ of 1053 g mol^−1^ (*M*_w_/*M*_n_=2.6). Such relatively low polymerization degrees (approx. 9) were additionally observed in previous investigations where formation of higher molecular weight materials proceeded in two competitive reaction pathways; that is, traditional cationic polymerization of the vinyl group and a polyaddition reaction favored by the ring closure of the hydroxybutyl ether.[^69^, ^70^, ^71^] A self‐polyaddition, favored by the structural pattern of **M1**, may explain the competition of this reaction in acidic environment since the reactivity of this conjugate acid‐initiated polymer formation proceeded with acceptable reaction rate comprising [(CF_3_SO_2_)_2_N]^−^. Onium salts comprising this anion show less reactivity in systems following rather a traditional cationic polymerization protocol where chain growth proceeds by the carbocation as intermediate. Oxiranes there typically serve as monomers.^[20]^ Here, the nucleophilicity of the anion appears too high to accomplish a highly reactive system in cationic polymerization since the higher nucleophilicity of this anion avoids an efficient chain growth according to a cationic polymerization mechanism. Obviously, the self‐polyaddition leads to the polymer shown in Scheme 4.[^70^, ^71^] It can obviously tolerate higher nucleophilicity of participating anions/species. This may have an impact on future developments in this field since poly(4‐hydroxy vinyl ether) may contribute as plasticizing agent in systems forming semi‐interpenetrating polymer networks based on radical polymerization using multifunctional (meth)acrylic esters and monomer **M1**.

A further point requires attention. The α‐hydrogen of the ether carbon possesses a lower C−H bond dissociation energy.^[72]^ Thus, an electrophilic radical formed in the initiation mechanism can easily abstract a hydrogen from there resulting in a nucleophilic radical; that is, CH_2_=CH−O−C^.^H−R. This intermediate should be easily oxidized by reaction with the onium salt resulting in formation of the respective cation CH_2_=CH−O−C^+^H−R as previously shown by alternative reactions with onium salts.^[73]^ It is stabilized by release of conjugate acid explaining the high reactivity of vinyl ethers in cationic polymerization.^[68]^ Our experimental results support these findings. Thus, exposure of **3 a** and **4** in the presence of **5** resulted in an increase of [*a* ${}_{H{}^{+}}$ ]/[**Sens**] to 0.41 and 0.33, respectively, exposing in CH_3_CN:**M1**=4:1 (vol %). This relates to an increase of 36 % in the case of **3 a** and 60 % in the case of **4** with respect to the data received without vinyl ether **M1**, Table 1. It also evidences the aforementioned hypothesis that nucleophilic radicals can react with **5** resulting in formation of conjugated acid.

In addition, the aforementioned carbocation formed can also competitively add **M1** resulting in branched structures (see Figures in SI showing that NMR spectra do not only comprise structural elements of Scheme 4 and shorter polymerization degree.

## Conclusion

Sensitizers **3 a** and **4** have brought new impetus in this field. Their electronic structure exhibits either an open and non‐bridged polymethine chain as in **3 a** or even a longer polymethine chain as in **4**, respectively. Surprisingly, **3 a** required a lower activation barrier in photoinitiated radical polymerization compared to derivatives exhibiting the same number of polymethine moieties; that is, **3 b**–**d**. Future developments may focus on the design of cyanine‐based sensitizers exhibiting a polymethine chain with no bridging moieties while availability at larger scale may give rise to further issues.

Structures **1** and **2** comprising a benzo[*cd*]indolium pattern showed good bathochromic shift of absorption on the one hand side but lower sensitization activity on the other hand. These compounds may become of interest to generate heat on demand just by turning on a light source in technologies where nowadays oven technologies operate either to initiate chemical reactions based on thermal activation or physical events such as removal of volatile components in coatings. Recent demands of the society to save energy and resources have enforced us to pursue such strategies. From this point of view, this contribution brings valuable aspects in this field regarding future design of cyanines for their use in photopolymer systems; that is, to connect them to photoinitiate either chemical events where **3** and **4** present acceptable structures or to photoinitiate physical events such as photonic drying with structures based on **1** or **2**.

Based on the results obtained with **4**, it would be interesting to study if incorporation of additional methine moieties, which would typically result in a significant bathochromic shift of absorption, will also enable excitation wavelengths around 1000–1100 nm. Two methine moieties typically result in a 100 nm bathochromic absorption shift. This spectral region still challenges to design cyanines showing sufficient **PET** at this spectral region. Nowadays, available compounds mainly generate heat upon excitation on demand and therefore function only as absorbers. Hopefully, the design of respective structures will bring additional impetus in the near future.

The general question will also arise about the practicability to access such structures. From this point of view, **3 b**–**d** provide easier access due to the availability of the connecting bridge available by Vilsmeier reaction. Here, new directions should come to develop alternative synthetic routes giving access to more structures with open polymethine chain absorbing around 1000 nm, keeping in mind the feasibility to access such structures and to receive materials resulting in acceptable shelf‐life in systems comprising iodonium salt and vinyl monomers.

## Conflict of interest

The authors declare no conflict of interest.

## Supporting information

As a service to our authors and readers, this journal provides supporting information supplied by the authors. Such materials are peer reviewed and may be re‐organized for online delivery, but are not copy‐edited or typeset. Technical support issues arising from supporting information (other than missing files) should be addressed to the authors.

Supporting Information
