# Supplementary material for: Rational Selection of Cyanines to Generate Conjugate Acid and Free Radicals for Photopolymerization upon Exposure at 860 nm
Source: Angew Chem Int Ed Engl. 2021 Oct 19;60(51):26855–65. doi: 10.1002/anie.202108713 (PMC9298067; doi:10.1002/anie.202108713)
Supplement: Supplementary file 1 — Supporting Information [file ANIE-60-26855-s001.pdf]

Angewandte Chemie
Eine Zeitschrift der Gesellschaft Deutscher Chemiker

Supporting Information

Rational Selection of Cyanines to Generate Conjugate Acid and Free Radicals for Photopolymerization upon Exposure at 860 nm

*Qunying Wang, Sergey Popov, Alfred Feilen, Veronika Strehmel, and Bernd Strehmel**

anie_202108713_sm_miscellaneous_information.pdf

Materials

Near-infrared sensitizers **1**, **2**, **3**, and **4** are commercially available. The respective trade names, their suppliers and CAS Numbers can be found in Table SI1. The iodonium salt bis-(4-t-butylphenyl)-iodonium bis(trifluoromethylsulfonyl)imide, commercially available as S2430 (**5**) from FEW Chemicals, was used as received. The monomer tri(propylene glycol) diacrylate (**TPGDA**) was purchased from Sigma-Aldrich. **TPGDA** (**M2**) was run through basic Al_2O_3 (Carl Roth GmbH) to remove the inhibitor before used for the polymerization, while all the other reagents were used without any further purification. Remaining materials used such as methanol, tetrahydrofuran (THF), acetonitrile, 1,4-butanediol vinyl ether and Rhodamine B lactone were purchased from Sigma Aldrich. Solvents used were spectroscopic grade.

Instrumentation and Procedures

The high-power NIR prototype emitting at 860 nm, of which the exposure intensity could reach to 1.0 W/cm^2 was received from EASYTEC GmbH. It was used to expose large areas. Due to its large size, another LED of smaller size emitting at 860 nm available from EASYTEC GmbH with an intensity of 1.0 W/cm^2 was only used for real time FTIR and photo-DSC. In addition, an LED exhibiting an emission maximum at 820 nm with the intensity of 1.0 W/cm^2 was available from Phoseon Ltd[SI1].

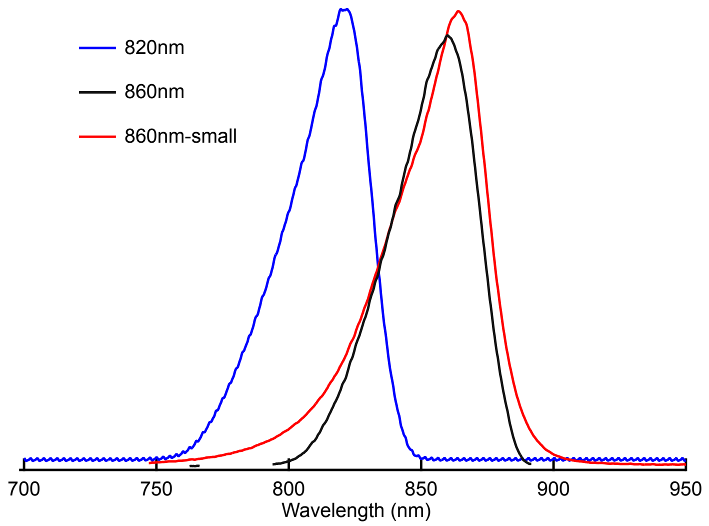

Figure SI1. High-power NIR-LED emission spectrum obtained by a fiber optical spectrometer from Ocean Optics (USB4000)

Table SI1: Summary of NIR sensitizers used

Abbr.	Trade name	Supplier	Name	CAS number
**1a**	S 0773	FEW Chemicals GmbH	1-Butyl-2-[3-(1-butylbenz[*cd*]indol-2(1*H*)-ylidene)-1-propenyl]-(*E*,*E*)-benzo[cd]indolium tetrafluoroborate	143185-79-9
**1b**	S08731	Spectrum Info, Ltd.	6-Butoxy-2-[3-(6-butoxy-1-butylbenz[*cd*]indol-2(1*H*)-ylidene)-1-propen-1-yl]-1-butyl- benzo[*cd*]indolium trifluorotris(1,1,2,2,2-pentafluoroethyl)phosphate	2459374-53-7
**2a**	S09426	Spectrum Info, Ltd.	1-Butyl-2-[5-(1-butylbenz[*cd*]indol-2(1*H*)-ylidene)-1,3-pentadien-1-yl]-benzo[*cd*]indolium tetrafluoroborate	1135933-68-4
**2b**	S 2437	FEW Chemicals GmbH	9-Butoxy-3-[(1E,3E)-5-[(3Z)-9-butoxy-2-butyl-2-azatricyclododeca-1(11),4(12),5,7,9-pentaen-3-ylidene]penta-1,3-dien-1-yl]-2-butyl-2-azatricyclododeca-1(11),2,4(12),5,7,9-hexaen-2-ium tetrafluoroborate	1135933-71-9
**3a**	S 2137	FEW Chemicals GmbH	1-Butyl-2-[7-(1-butyl-3,3-dimethyl-1,3-dihydro-indol-2-ylidene)-hepta-1,3,5-trienyl]-3,3-dimethyl-3H-indolium hexafluorophosphate	134339-08-5
**3b**	S 2026	FEW Chemicals GmbH	1-Butyl-2-(2-[3-[2-(1-butyl-3,3-dimethyl-1,3-dihydro-indol-2-ylidene)-ethylidene]-2-phenyl-cyclohex-1-enyl]-vinyl)-3,3-dimethyl-3H-indolium 4-methylbenzenesulfonate	1628790-42-0
**3b-ox**	S 2178	FEW Chemicals GmbH	1-Butyl-2-[2-[3-[2-(1-butyl-1,3-dihydro-3,3-dimethyl-2*H*-indol-2-ylidene)ethylidene]-2-phenyl-1,4-cyclopentadien-1-yl]ethenyl]-3,3-dimethyl-, 3*H*-Indolium, 4-methylbenzenesulfonate	2434784-87-7
**3c**	S10761	Spectrum Info, Ltd.	2-[2-[3-[2-(1,3-Dihydro-1,1,3-trimethyl-2*H*-benzo[*e*]indol-2-ylidene)ethylidene]-2-(diphenylamino)-1-cyclopenten-1-yl]ethenyl]-1,1,3-trimethyl-, (*OC*-6-22)1*H*-benzo[*e*]indolium, trifluorotris(1,1,2,2,2-pentafluoroethyl)phosphate	2561240-74-0
**3d**	S 2025	FEW Chemicals GmbH	1-Butyl-2-(2-[3-[2-(1-butyl-3,3-dimethyl-1,3-dihydro-indol-2-ylidene)-ethylidene]-2-diphenylamino-cyclopent-1-enyl]-vinyl)-3,3-dimethyl-3H-indolium tetrafluoroborate	1888324-74-0
**4**	S09442	Spectrum Info, Ltd.	2-[(1*E*,3*E*)-3-[3-[(1*E*,3*E*)-3-(1,3-Dihydro-1,3,3-trimethyl-2*H*-indol-2-ylidene)-1-propen-1-yl]-5,5-dimethyl-2-cyclohexen-1-ylidene]-1-propen-1-yl]-1,3,3-trimethyl-3*H*-Indolium trifluorotris(1,1,2,2,2-pentafluoroethyl)phosphate(1-)	2319601-73-3

Cyclic voltammetry

We followed a previous protocol to obtain oxidation and reduction potentials of the sensitizers[SI2]. Thus, cyclic voltammetry (pursued with a Versastat4-400 from AMETEK served as potentiostat) was carried out to measure the oxidation and reduction potential of the aforementioned sensitizers (10^{-3} mol/L) with tetrabutylammonium hexafluorophosphate (0.1 mol/L) from Sigma Aldrich as a supporting electrolyte while ferrocene served as an external standard in acetonitrile. All the data were collected at a scanning rate of 0.015 V/s by using platinum disc as a working electrode and Ag/AgCl as reference electrode[SI2].

Fluorescence emission maximum (λ^{f}_{max}) *fluorescence decay time* (τ_f) *and quantum yield* (Φ_f)

The regular fluorescence set up (FluoTime 300 from PICOQUANT) was carried for recording fluorescence emission λ^{f}_{max}, to take decay time τ_f, and quantum yield Φ_f with the excitation from laser (LDH-P-C-670) until 910 nm. Time correlated single photon counting was the technique to take the emission decay. This instrument from picoquant provides the opportunity to measure decay between 25 ps and several milliseconds. The instrument was equipped with high resolution emission double monochromator operated in subtractive mode (2x 300 mm focal length, UV/VIS/IR grating pair 1st stage: 600 l / mm blaze 1250 nm and 1200 l / mm blaze 500 nm, dispersion: 4.5 nm / mm, UV / VIS grid pair 2nd stage: 1200 l / mm blaze 500 nm 1200 l / mm blaze 500 nm (-), 2.7 nm / mm (-), PMA-C 192-M Cooled photomultiplier) for the emission side. Excitation proceeded by using a Computer controlled diode laser driver for picosecond pulses (196 kHz and 80 MHz, external trigger input). The system can be also operated in *cw*-mode to record spectra in additive mode (monochromator: blaze 500 nm (+), dispersion: 1,4 (+)). The diode laser used operated at 677 nm. EasyTau software and laser control software controlled the system. This also facilitated to calculate decay time by the software by iterative convolution. An appropriate scatter (Ludox in water) was taken to record the instrumental response function of the system. Time resolution of the instrument is >25 ps. For preparation, the sample was dissolved in methanol (spectroscopic grade) to get the absorption of extinction of < 0.1 at 677 nm. The EasyTau Software also operated to calculate the

decay time by iterative convolution between the instrumental response and the sample.

Absolute fluorescence quantum yields were measured with an integrating sphere provided by PICOQUANT for the FluoTime 300 spectrometer. First the respective counts available by the laser were measured using a blanc cuvette comprising spectroscopic grade solvent. Then, the sample was changed with a cuvette comprising the same solvent and the absorber exhibiting an absorption between 0.05-0.1 at 677 nm. This treatment resulted in the absorption of the absorber taken for measurement needed to determine luorescence quantum calculated with the EasyTau software that also controlled the instrument.

Fluorescence quantum yields of **1b**, **2a**, **3c**, and **4** were taken by a FS 920 from Edinburgh Instruments by use of a reference. This was carried out with Sulforhodamine 101[SI3] applying correction mode for both excitation and emission using 1 cm cuvette at 20-22°C. More details can be found elsewhere[SI4]. GMBU (Jena) took these measurements on request as service to provide data for this contribution that resided mainly outside of the spectral response or the FluoTime 300 from Picoquant.

Nuclear Magnetic Resonance (NMR)

A Fourier 300 serves as source to take the ^{1}H-NMR spectra to explore the reaction of **M1** (1,4-butanediol vinyl ether). The system comprising **4** (0.005 mmol/g) and **5** (0.03 mmol/g) in **M1** was exposed in a round glass dish (height: 1cm, diameter: 3cm) for 10 min applying an 860 nm LED-array (power: 1.0 W/cm^2, exposed area: 3 cm × 12 cm) for excitation. This was done under continuous nitrogen purging. 20 mg of the product obtained was dissolved in 1mL deuterated acetone for NMR measurement. As a comparison, the measurement from the system exposed without nitrogen purge and pure **M1** were additionally taken, respectively.

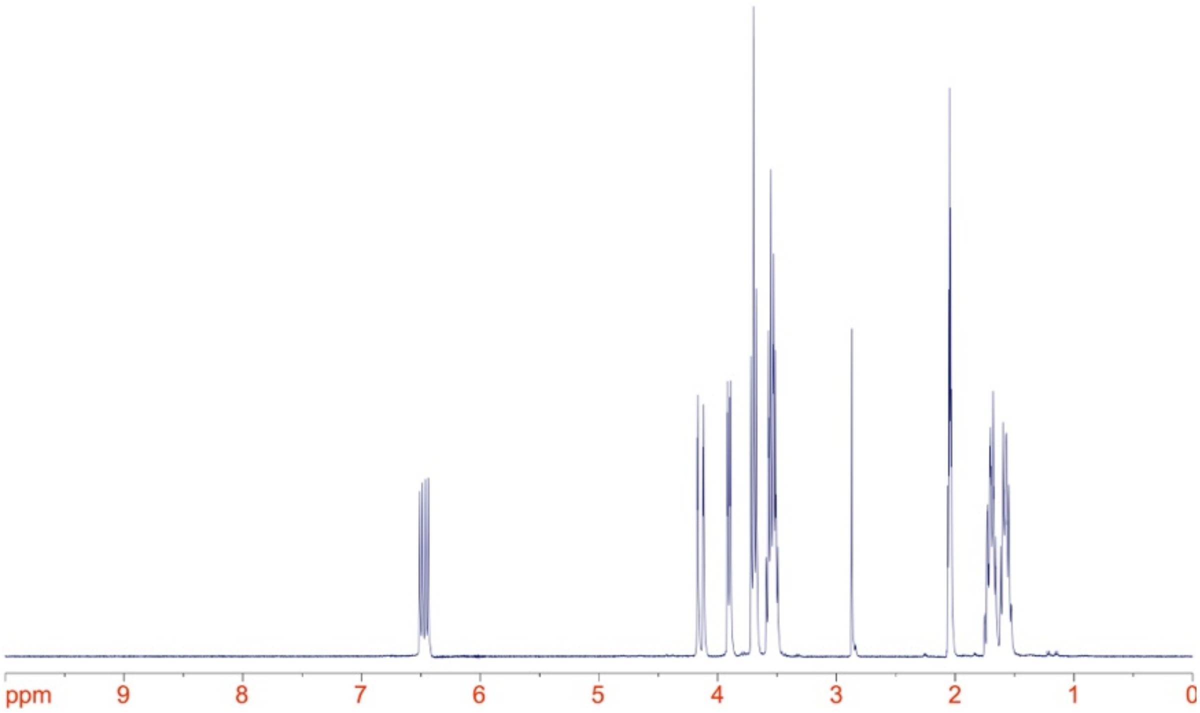

Figure SI2: M1 before exposure

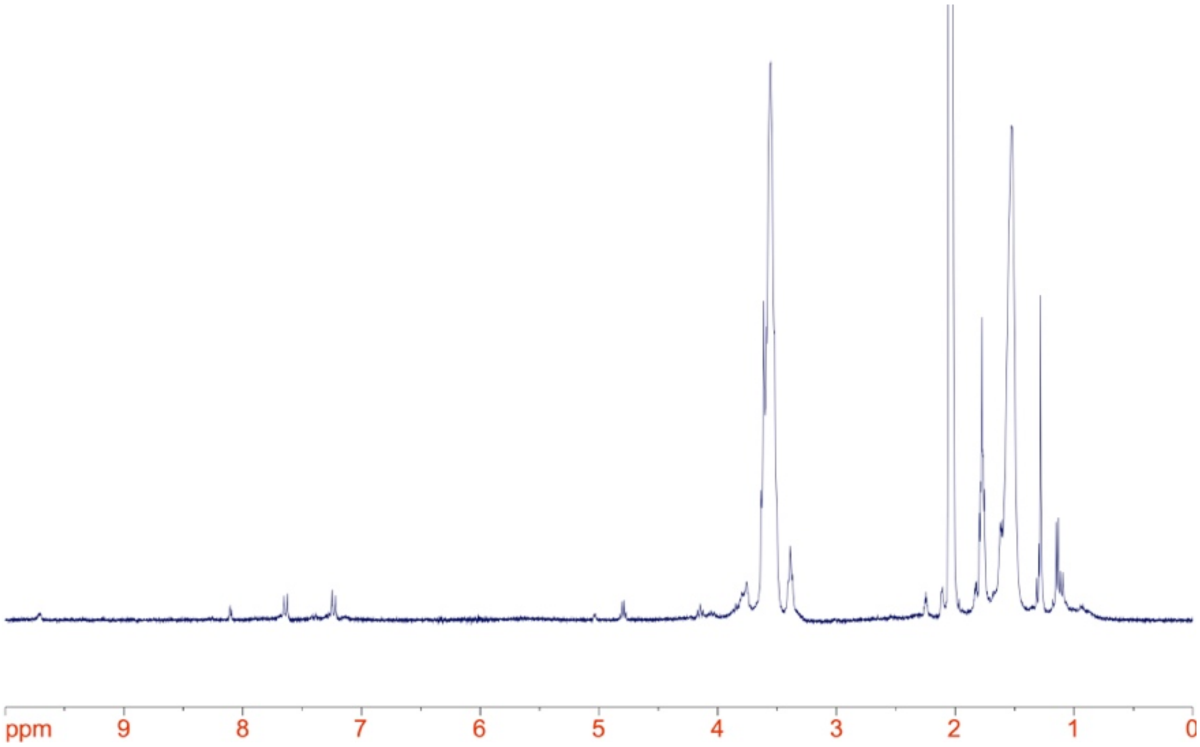

Figure SI3: M1 after exposure under air

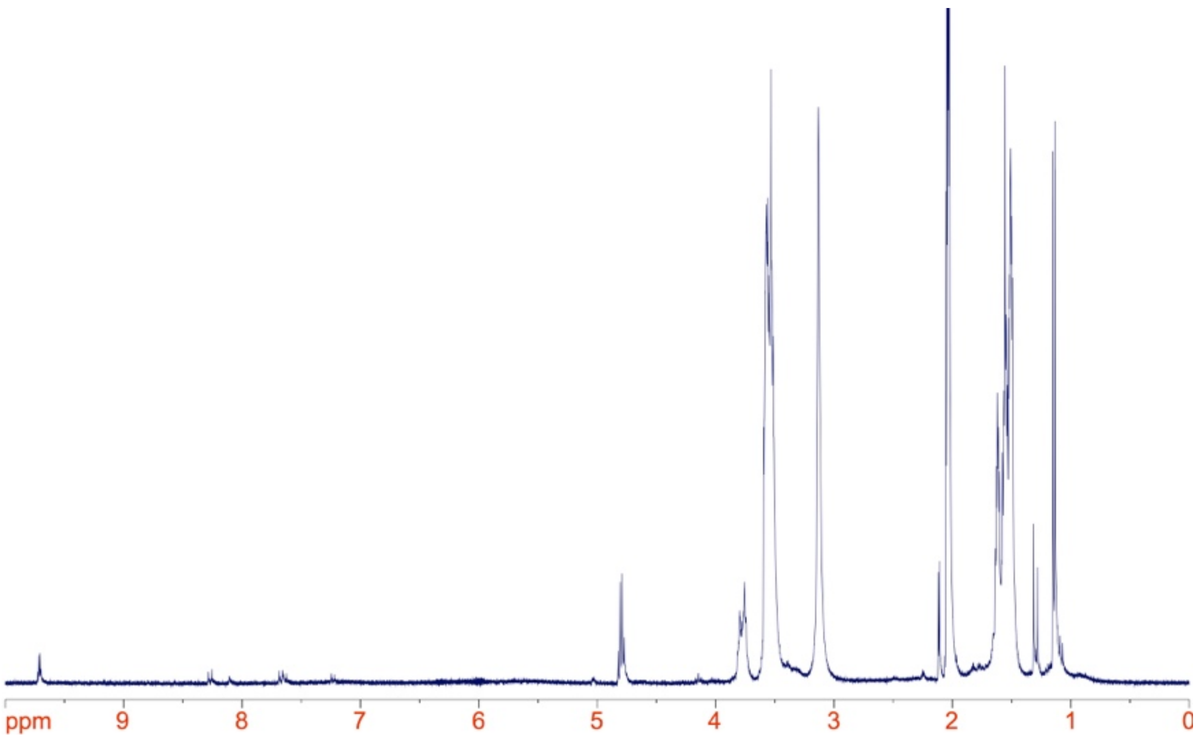

Figure SI4: M1 after exposure under nitrogen

GPC measurement

10 mg of the material made for the NMR measurement were additionally dissolved in 2 mL THF and transferred to GPC measurement. This was conducted with a GPC Viscotek 270 max using TGuard Col 10 x 4.6 mm and two T6000M General Mixed 3000 x 7.8 mm columns, a column temperature of 30 °C, an RI detector, and THF as an eluent at a flow rate of 1 mL/min. The column system was calibrated with 7 linear poly(methyl methacrylate) standards received from Shodex (1850 $g·mol^{-1}$; 6380 $g×mol^{-1}$; 20100 $g×mol^{-1}$; 73200 $g×mol^{-1}$; 218000 $g×mol^{-1}$; 608000 $g×mol^{-1}$; and 1050000 $g×mol^{-1}$). GPC data were analyzed using Omni SEC 4.6.2:GPC.

Photo-bleaching by UV-Vis.

The spectra of the dissolved sensitizers in the solvent were detected by the device with Evolution 220 from Thermo Scientific in 1×1 cm quartz cuvette. And the bleaching rate of the sensitizers were determined from the UV-Vis-NIR spectra before and after exposure. The acetonitrile solutions containing sensitizers ($5.0×10^{-6}$ mol/L) and iodonium salt of **5** ($3.0×10^{-5}$ mol/L) were irradiated in the covered cuvettes under air with an interval at 5s, 15s, 30s, 60s, 120s, 300s and 600s with the LEDs both 820 nm and 860 nm due to their absorption, respectively. As a comparison, the solutions with the same concentrated sensitizers (**1b**, **2a**, **3c** and **4**) in the absence of iodonium salt were also measured through the same procedure. The bleaching rate of the sensitizers also corresponds to the absorbance change of the sensitizers.

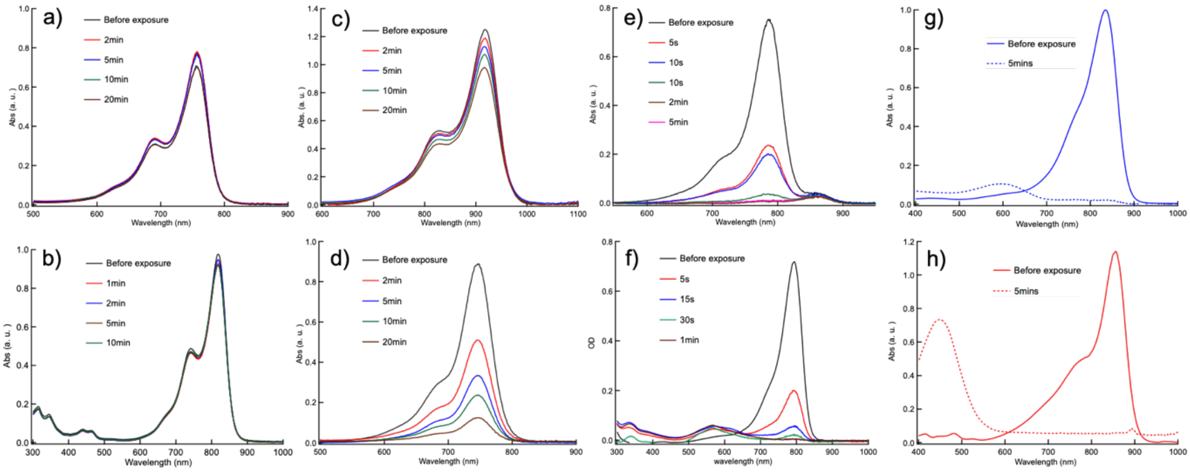

Figure SI5. Photo bleaching shown by decrease of extinctions of NIR-sensitizers comprising **5** in acetonitrile using the LEDs at 820 nm and 860 nm with the intensity of 1 W/cm^2 due to their absorption, respectively. a) **1a**, b) **1b**, c) **2b**, d) **3a**, e) **3b** and f) **3d**. Photo bleaching in the absence of 5 with N2 purge with 860 nm LED g) **3c** and h) **4**.

Photo-DSC

A regular photo-DSC setup[SI5] (Q2000 from TA Instruments) was applied to record the heat flux generated by polymerization of the monomer. This experiment operated under isothermal conditions. Thus, heat formed would not be available to activate chemical reactions. It offers therefore the possibility to study the response of the system at different temperatures still under isothermal conditions providing additional heat needed in order to initiate the polymerization at a temperature above a certain threshold. Below of this threshold, nearly no polymerization or very inefficient initiation shall proceed. The NIR light applied for irradiation was collected by a lens and projected into a two-arm fiber connected to the oven of the DSC device. Here, the intensity of the output light through the fiber arms was adjusted by a USB 4000 spectrometer operating as radiometer. In addition, a shutter was introduced to synchronize the light source and measurement placed between the light source and the lens. In order to investigate the reactivity of sensitizers, **3a** (0.001mmol/g) was together with **5** (0.015mmol/g) was first dissolved in **M2 (TPGDA**) for photo-DSC procedure at different temperature (25°C, 40°C and 60°C). This was carried out with NIR-LED emitting at 770nm at an intensity of 45 mW/cm^2 matching well with the absorption of **3a**. The same setup was applied to the system where **3c** or **4** operated as sensitizer under the aforementioned conditions taking the same concentrations. It required also to change the NIR-LED to receive optimal exposure conditions to study these both sensitizers. Here, a high-power LED with an output of 350 mW/cm^2 (fiber output) emission at 820 nm was used for **3a** exposure while a NIR-LED with an output of 200 mW/cm^2 at 860 nm (fiber output) served as exposure source to expose **4**, respectively. A USB 4000 served as radiometer to control the light intensity.

Real time Fourier-Transform Infrared Spectroscopy (FTIR)

Real time FTIR (Vertex 70 from Bruker) in an attenuated total reflection (ATR) mode was applied to investigate the kinetics of the photopolymerization where each data point was collected every 0.2 s. More details can be found in a previous report[SI1]. The initiator **5** was added into **M1** resulting in a concentration of 0.03 mmol/g following with adding of sensitizer (0.005 mmol/g) to the solution. Photopolymerization occurred upon the exposure of small-sized 860nm LED with an intensity of 1.0 W/cm^2. in addition, the sample thickness was controlled into around 30 μm and covered with

microslides to get rid of atmosphere air. For the kinetic evaluation, the wavenumber of C=C at 812 cm^{-1} was taken to calculate the conversion of the polymerization of **M2** (reference vibration: C=O at 1725 cm^{-1}). The vibration at 1615 cm^{-1} was applied to follow polymerization of the vinyl ether **M1** (refence band: 1205 cm-1 for C-O vibration).

Quantification of conjugate acid

The conjugate acid generated during exposure was quantified following a previous protocol[SI2]. A UV-Vis-NIR device (Evolution 220 from Thermo Scientific) monitored spectral changes caused by protonation of Rhodamine B lactone serving as optrode. Its protonation resulted in formation of red colored Rhodamine B exhibiting an absorption maximum at 550 nm. Acetonitrile served as solvent. For the procedure, the acetonitrile solution comprising the sensitizer (2.0×10^{-5} M) and iodonium salt (1.2×10^{-4} M) were dissolved in a volumetric flask of 10 mL and exposed in a cuvette (width of 1.0 cm) applying NIR-LED of either 860 nm or 820 nm for 15 mins for the respective sensitizers. Some acetonitrile was refilled to compensate evaporation loss of the solvent during the radiation. Afterwards UV-Vis. spectra of the solution aforementioned was collected following with addition of rhodamine B lactone (1.0×10^{-4} mol/L) and carried out with UV-Visible measurement. Background was subtracted accordingly[SI2]. In addition, the influence from **M1** for the formation of conjugate acid from **1b**, **2a**, **3c** and **4** is also investigated via the same method, in which 20% **M1** was introduced into the system. The conjugate acid formed quantified by rhodamine B lactone is to prove the existence of the acidic species through the exposure but not comparable to the active cation generated during the photopolymerization. While the reactivity tendency from different sensitizer with different pattern is comparable from this method.

Scheme SI1 shows the respective ring opening of Rhodamine B lactone (**RHB-L**) of conjugate acid (con-H+) resulting in colored Rhodamine B (**RHB-L**).

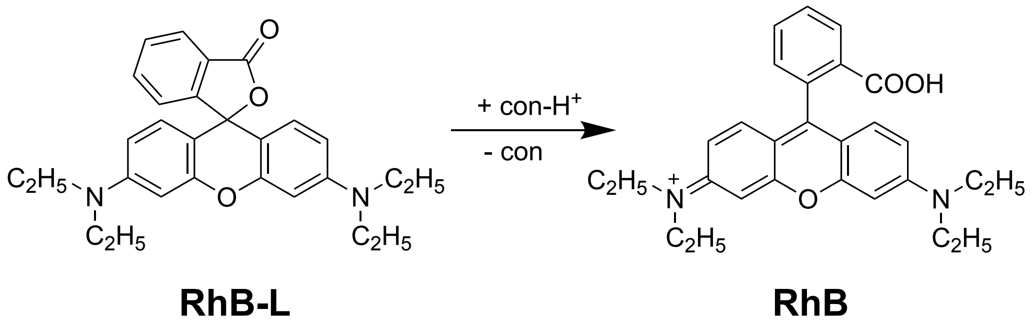

Scheme SI1

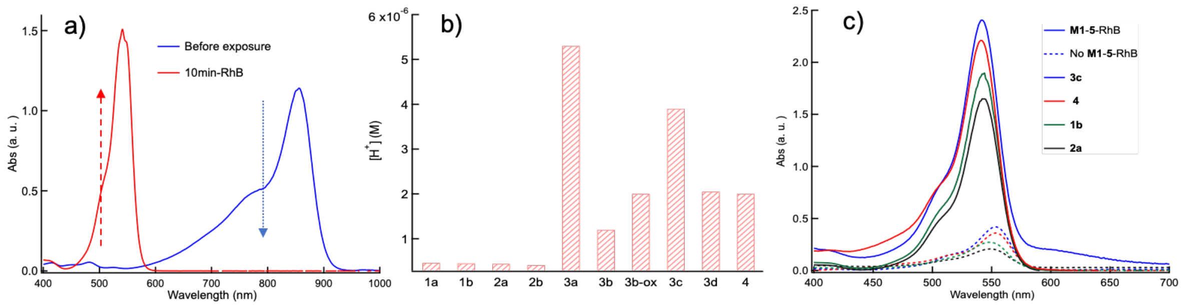

Figure SI6. a) Scheme for conjugate acid formation measurement from Sens **4**, b) conjugate acid formed from the sensitizers in the presence of **5** after exposure for 15 mins and c) UV-Vis. spectra shown the difference from the conjugate acid formation with and without **M1** from the sensitizers **1b**, **2a**, **3c** and **4**.

Liquid Chromatography-Mass Spectrometry (LC-MS)

The products of photoreaction were detected via a QTOF-LCMS system (G6530B) from Agilent where Dual AJS ESI was used as the ion source[SI1]. A column Hypersil C4 (125 x 4mm) from Thermofischer Scientific was used with acetonitrile/water (80:20) as dilution solvent, which was changed to 95:5 after 20 minutes. The photoproducts of the sensitizers obtained after exposure were analyzed. The photoreaction occurred in acetonitrile comprising sensitizer (2.0×10^{-5} M) with iodonium salt (1.2×10^{-4} M) upon the NIR-LED prototype at 860nm in the atmosphere.

Table SI2: Summary of molecular ions of photoproducts detected exposed a solution comprising S09442 and S2430 exposed at 860nm with an intensity of 1.2 W/cm^2 for 10min. Some of the ions appeared as doubly charged ions and/or NA^+-adducts

t/min	m/z
2,35	214,0860
2,72	214,0860
12,01	214,0860
12,22	214,0860
12,82	279,1547
13,37	352,2404
13,47	284,3265
14,26	427,2403
15,44	338,3364
15,64	803,5300
16,07	566,3827
16,66	561,4208
16,96	537,4220
17,61	565,4525
19,21	663,4424
21,07	896,7540
21,49	872,7559
21,76	872,7535
22,26	848,7519
23,98	876,7864
24,49	130,1569

Quantum chemical calculation

All calculations were performed with Spartan 16. Geometry of each sensitizer was optimized with the method B3LYP//6-31G* until a minimum was obtained. Frequency calculations indicated no negative values.

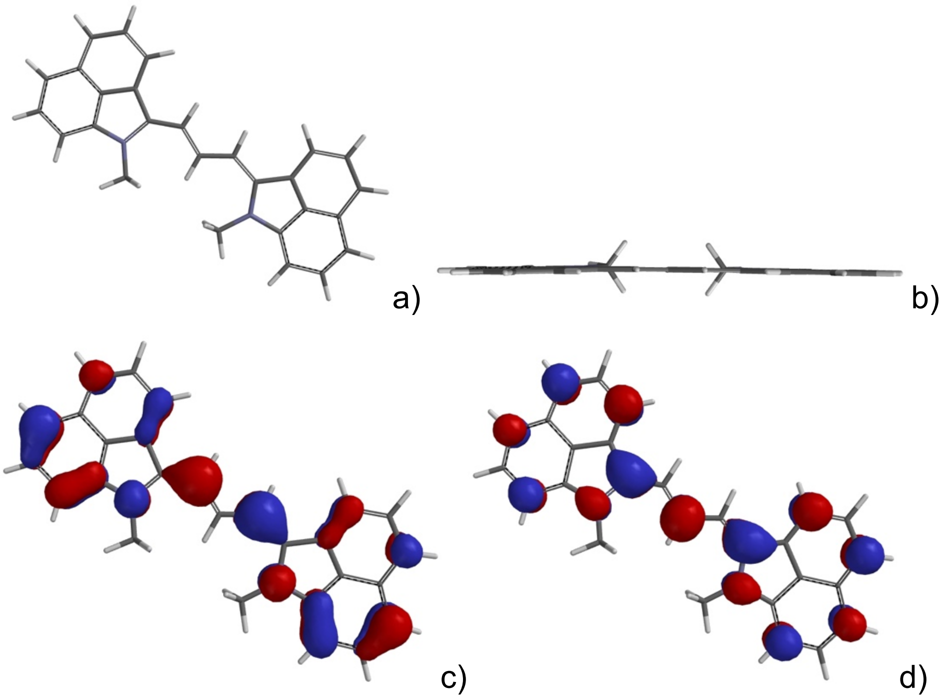

Figure SI7: Geometry of **1a** with a) front view and b) side view showing a nearly planar pattern. Slides c) and d) depict HOMO and LUMO, respectively.

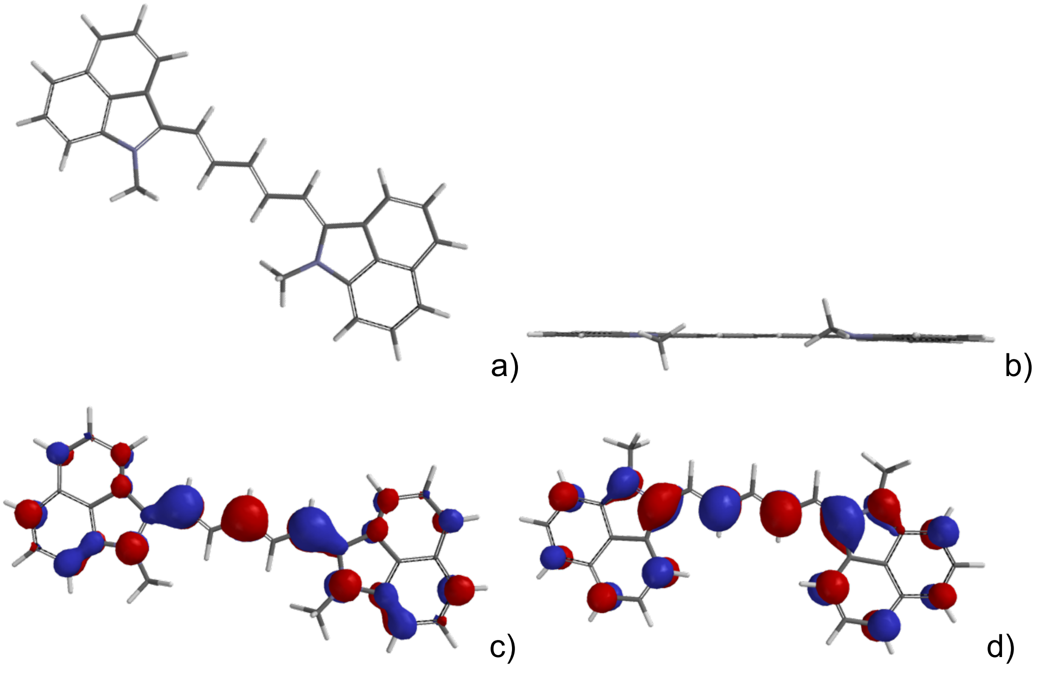

Figure SI8: Geometry of **2a** with a) front view and b) side view showing a nearly planar pattern. Slides c) and d) depict HOMO and LUMO, respectively.

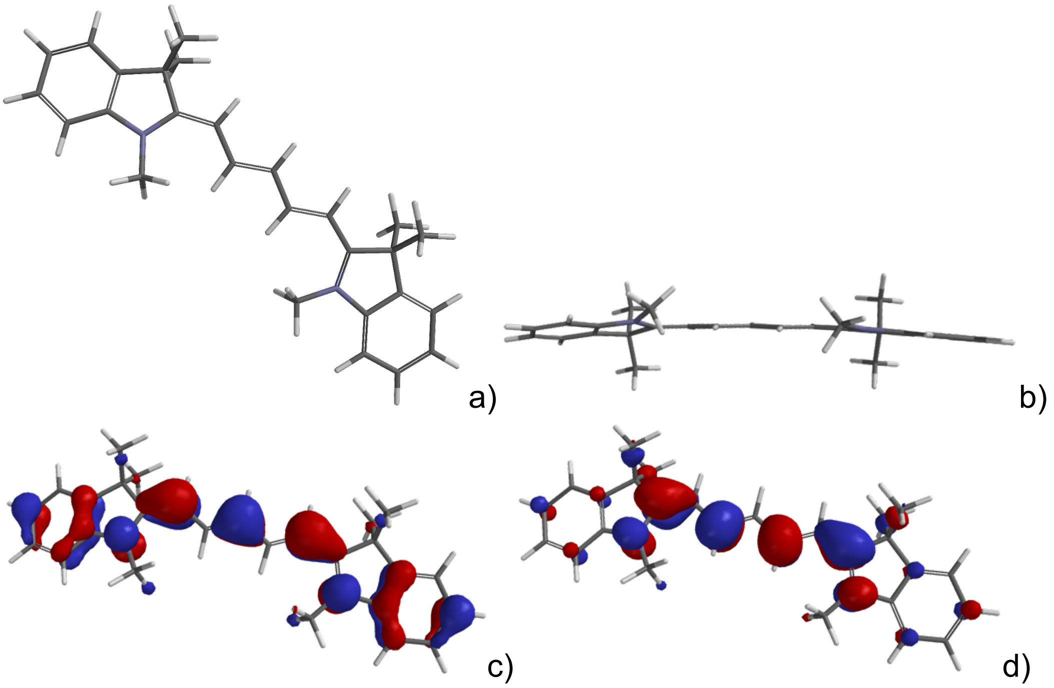

Figure SI9: Geometry of **3a** with a) front view and b) side view showing a nearly planar pattern. Slides c) and d) depict HOMO and LUMO, respectively.

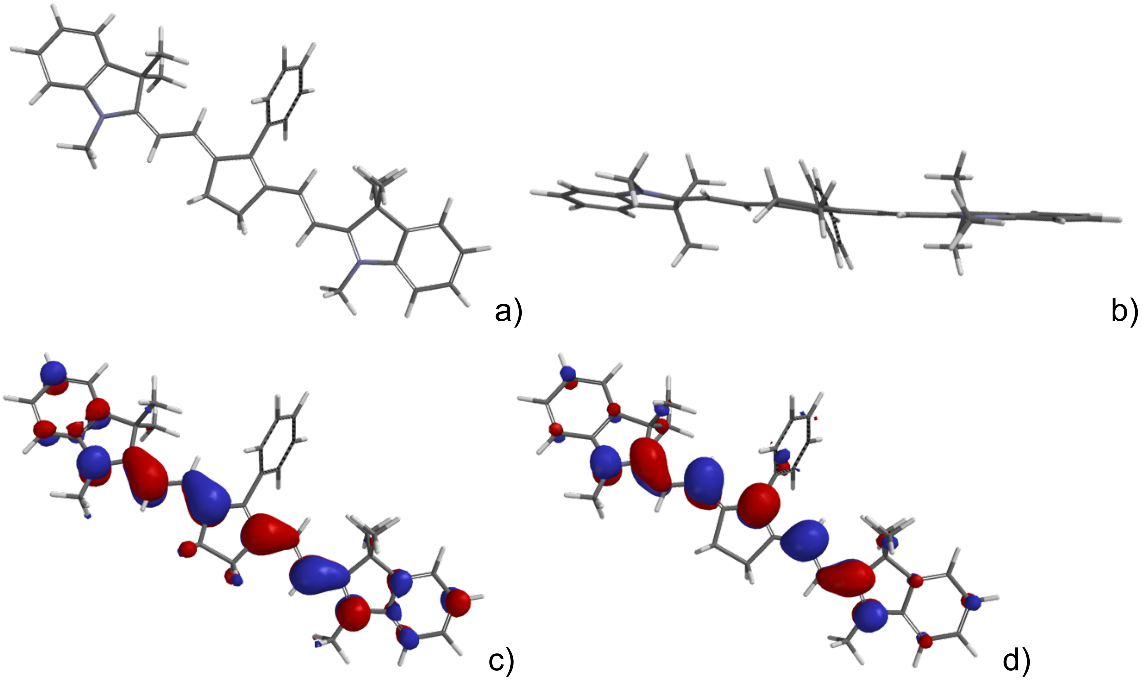

Figure SI10: Geometry of **3b** with a) front view and b) side view showing a nearly planar pattern. Slides c) and d) depict HOMO and LUMO, respectively.

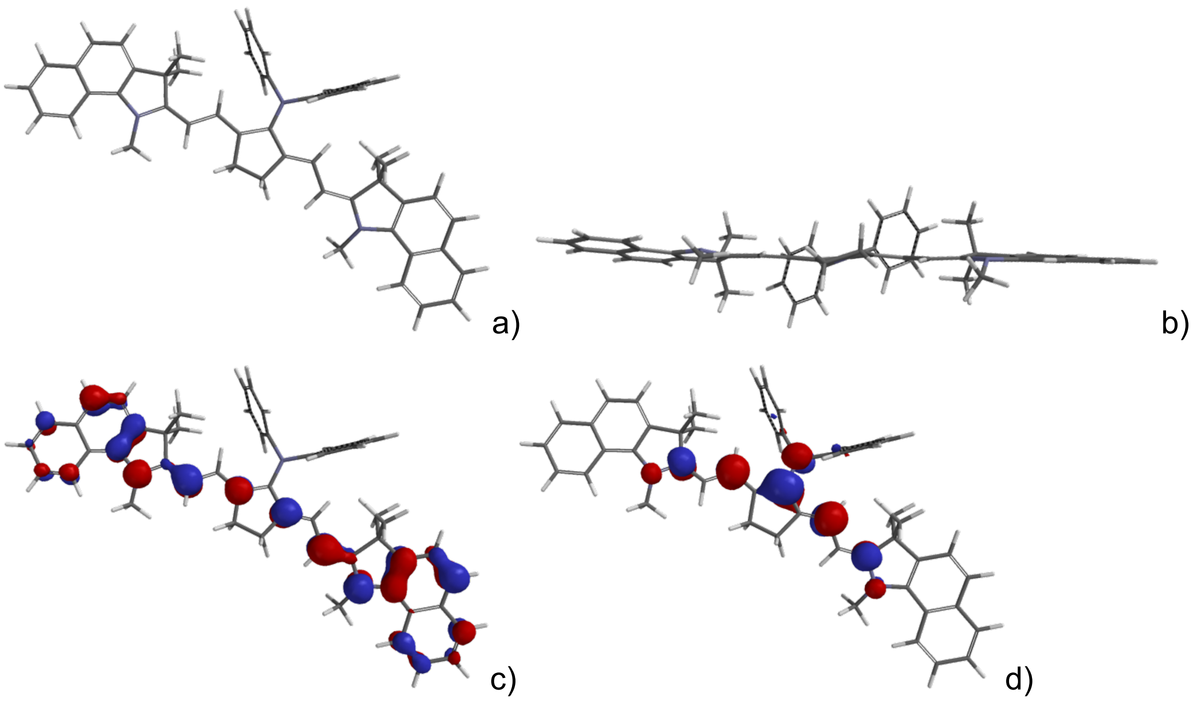

Figure SI11: Geometry of **3c** with a) front view and b) side view showing a nearly planar pattern. Slides c) and d) depict HOMO and LUMO, respectively.

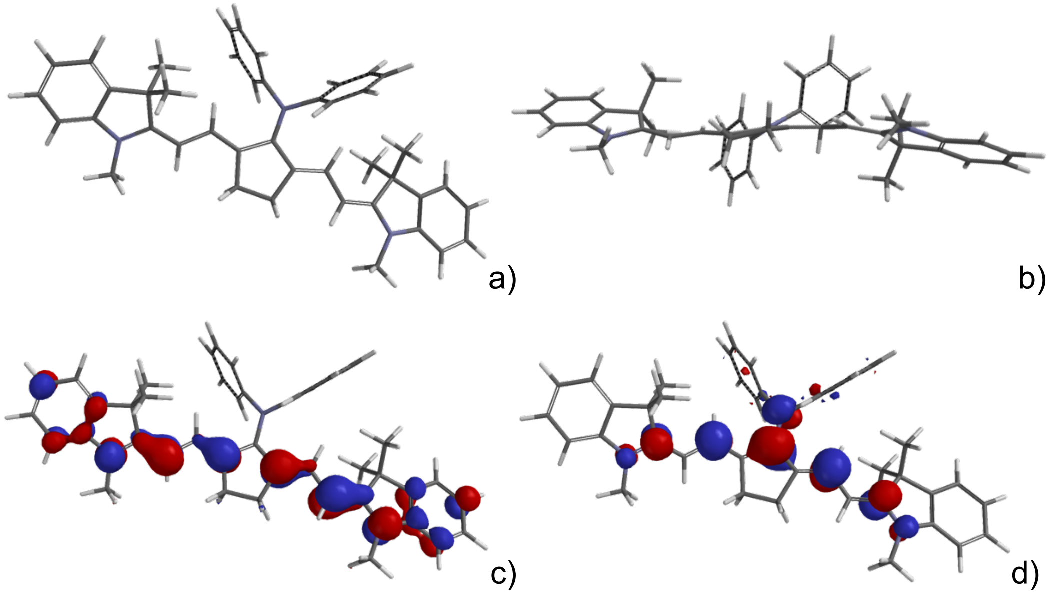

Figure SI12: Geometry of **3d** with a) front view and b) side view showing a nearly planar pattern. Slides c) and d) depict HOMO and LUMO, respectively.

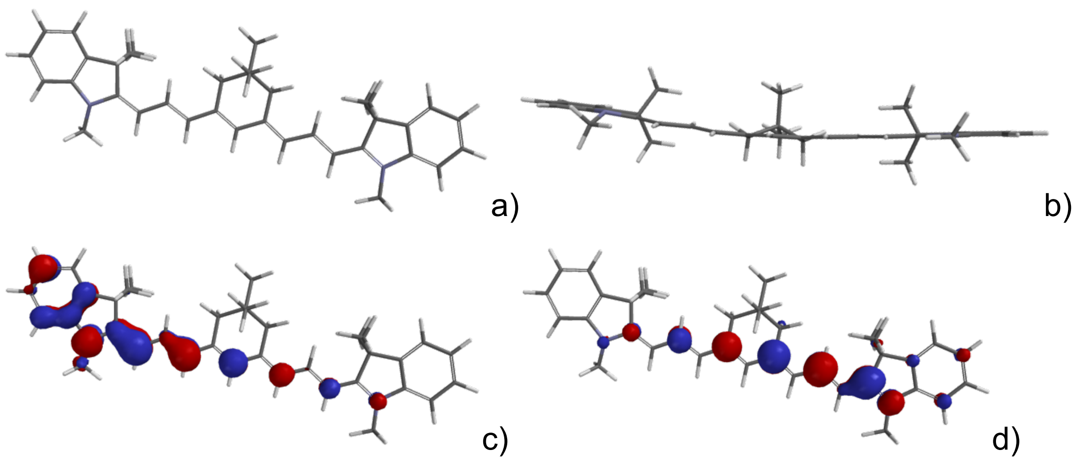

Figure SI13: Geometry of **4** with a) front view and b) side view showing a nearly planar pattern. Slides c) and d) depict HOMO and LUMO, respectively.

Temperature evolution

A NIR sensitive camera (test 0563 0885 V7) was used to measure the temperature generate by sensitizers in the process of the NIR irradiation. Details were previously disclosed[SI6-8].

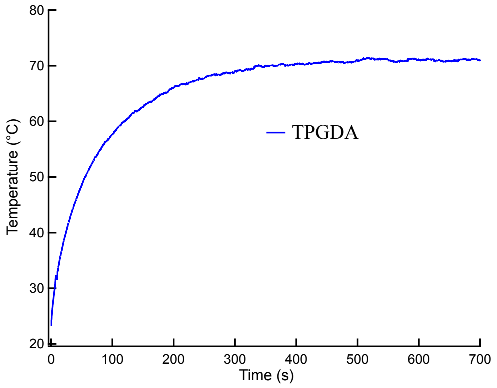

Figure SI14: Temperature evolution of a TPGDA matrix embedded with **1b** upon exposure with 860 nm NIR-LED device (I=1 W/cm^2)

<u>*References*</u>

SI1 C. Schmitz, Y. Pang, A. Gülz, M. Gläser, J. Horst, M. Jäger, B. Strehmel, *Angew. Chem., Int. Ed.* **2019**, *58*, 4400-4404.

SI2 C. Schmitz, A. Halbhuber, D. Keil, B. Strehmel, *Progress in Organic Coatings* **2016**, *100*, 32-46.

SI3 K. Rurack, M. Spieles, *Analytical Chemistry* **2011**, *83*, 1232-1242.

SI4 B. Strehmel, C. Schmitz, C. Kütahya, Y. Pang, A. Drewitz, H. Mustroph, *Beilstein Journal of Organic Chemistry* **2020**, *16*, 415-444.

SI5 T. Brömme, D. Oprych, J. Horst, P. S. Pinto, B. Strehmel, *RSC Advances* **2015**, *5*, 69915-69924.

SI6 C. Schmitz, Y. Pang, A. Gülz, M. Gläser, J. Horst, M. Jäger and B. Strehmel, *Angew. Chem., Int. Ed.*, 2019, **58**, 4400-4404.

SI7 Y. Pang, S. Fan, Q. Wang, D. Oprych, A. Feilen, K. Reiner, D. Keil, Y. L. Slominsky, S. Popov, Y. Zou and B. Strehmel, *Angew Chem Int Ed Engl*, 2020, **59**, 11440-11447.

SI8 Y. Pang, A. Shiraishi, D. Keil, S. Popov, V. Strehmel, H. Jiao, J. S. Gutmann, Y. Zou and B. Strehmel, *Angew Chem Int Ed Engl*, 2021, **60**, 1465-1473.
